# Cell-Instructive Surface Gradients of Photoresponsive Amyloid-like Fibrils

**DOI:** 10.1021/acsbiomaterials.1c00889

**Published:** 2021-09-13

**Authors:** Adriana Maria Ender, Kübra Kaygisiz, Hans-Joachim Räder, Franz J. Mayer, Christopher V. Synatschke, Tanja Weil

**Affiliations:** Department Synthesis of Macromolecules, Max Planck Institute for Polymer Research, Ackermannweg 10, 55128 Mainz, Germany

**Keywords:** peptide amyloid fiber, MALDI-MSI, bioactive gradient, photocontrolled cell adhesion

## Abstract

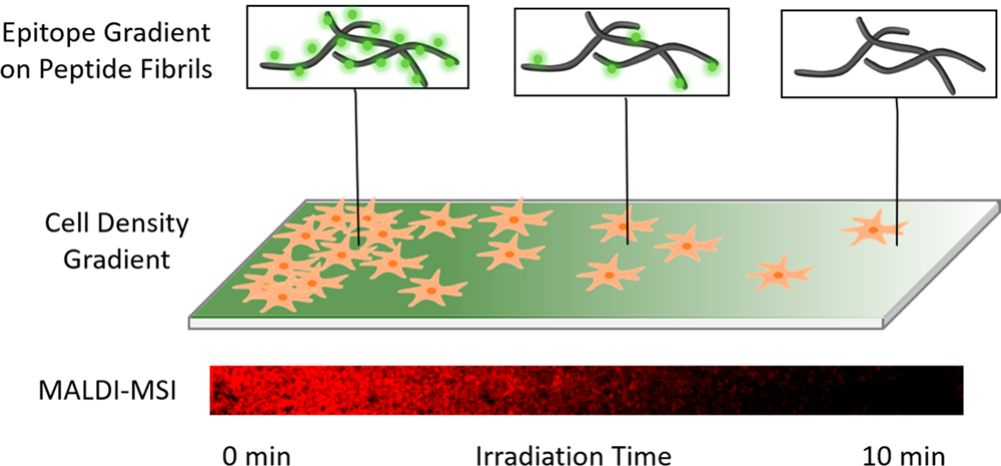

Gradients of bioactive molecules play a crucial role in various biological processes like vascularization, tissue regeneration, or cell migration. To study these complex biological systems, it is necessary to control the concentration of bioactive molecules on their substrates. Here, we created a photochemical strategy to generate gradients using amyloid-like fibrils as scaffolds functionalized with a model epitope, that is, the integrin-binding peptide RGD, to modulate cell adhesion. The self-assembling β-sheet forming peptide (CKFKFQF) was connected to the RGD epitope via a photosensitive nitrobenzyl linker and assembled into photoresponsive nanofibrils. The fibrils were spray-coated on glass substrates and macroscopic gradients were generated by UV-light over a centimeter-scale. We confirmed the gradient formation using matrix-assisted laser desorption ionization mass spectroscopy imaging (MALDI-MSI), which directly visualizes the molecular species on the surface. The RGD gradient was used to instruct cells. In consequence, A549 adapted their adhesion properties in dependence of the RGD-epitope density.

## Introduction

Concentration gradients in the physiochemical environment of the extra cellular matrix (ECM) play a crucial role for cell adhesion and growth.^1,2^ Gradual alterations of biochemical signals are the driving force for events like directed cell migration, that is, during nerve- and skin regeneration, vascularization, and immune responses.^3,4^ Since these cell-material interactions occur at the mesoscopic length scale, and the ECM is composed of a dense network of fibrillar structures,^5−8^ self-assembling peptides that form fibrils are promising biomaterials serving as scaffolds for cellular growth, adhesion, spreading and migration.^9,10^

Amyloid-forming peptides are a special group of peptides assembling into highly ordered fibrils with a characteristic cross β-sheet structure and characteristic physical properties such as long-term stability in physiological environments, mechanical stiffness, and strong adhesion to various substrates.^11^ Many amyloid-forming peptides also exhibit intrinsic bioactivity and have recently evolved from a class exclusively associated with pathology^12,13^ to functional materials^14,15^ with applications, such as stimulating nerve growth for tissue engineering^16−18^ and increased retroviral cell uptake for gene therapy.^19,20^ In Nature, the intrinsic adhesiveness and high aspect ratio of amyloid fibrils provide structural integrity to bacteria biofilms^21,22^ and allow material-efficient substrate coverage.

Therefore, amyloid-like peptides featuring these favorable nanomechanical properties, as well as an intrinsic bioactivity, could be appropriate scaffolds for mimicking concentration gradients of the ECM.^23^ Functionalization of the amyloid-scaffold, for example, with certain ECM protein-derived epitopes, such as the laminin-derived peptide sequence RGD can further increase their bioactivity.^5,24^ The RGD-motif is involved in regulating several cellular processes, such as cell attachment, spreading, orientation, proliferation, differentiation, and even directional cell migration, and it is, therefore, widely applied to facilitate cell–substrate interactions.^4,25^

Several examples of RGD-carrying amyloids have been reported in the literature. For example Gras et al. could alter cell attachment^26^ and cell compatibility^27,28^ on coatings from RGD-modified YTIAALLSPYS peptide.^24^ Neuronal cells in culture also benefited from incorporating the RGD motif into amyloids in terms of cell attachment and neurite outgrowth.^9,29^ The accessibility of the RGD motif is an important parameter when designing a self-assembled nanomaterial. This was studied in a combined theoretical and experimental study for different RGD-carrying hexapeptides.^30^ Supramolecular coassembly of nonfunctional and RGD-modified peptides provides easy access to bioactive materials with adjustable epitope concentrations as demonstrated for peptide hydrogels.^31^

While fibrous scaffolds are versatile supramolecular biomaterials, very few examples for molecular gradients within this material class exist.^32^ Contemporary strategies to create molecular gradients on surfaces that can direct or guide cellular behavior^4,33,34^ include bipolar electrochemistry, microfluidic systems, and dip coating techniques.^35,36^ However, many of these techniques require a laborious setup and create large gradient sizes in the millimeter regime.^35−37^ In contrast, photoreactive chemistry is an extremely versatile tool providing high spatial resolution only limited by the wavelength of light^38^ and experimental setup, for example, by a photomask.^39,40^ Photoinduced spatial release of bioactive substances^41^ and precise positioning of molecules and cells on various surfaces has been achieved and applied down to a submicrometer level.^5,42−45^ Nitrobenzyl esters are well established as a class of light-responsive groups that undergo a photocleavage reaction upon irradiation with UV light. For example, Del Campo et al. and Wegner et al. created patterned cell distributions on coatings of covalently bound bioactive molecules on glass surfaces that were photoreleased on demand.^42,44^ Stupp et al. were able to control the bioactivity of peptide amphiphile nanofibers by using the nitrobenzyl group as a light-sensitive linker to remove attached RGDS-epitopes from fiber surfaces.^5^ Furthermore, by using the nitrobenzyl as a caging group, Yousaf et al. accomplished a gradual distribution of RGD (Arg-Gly-Asp) bound to gold-coated glass surfaces, which resulted in signal-driven cell migration.^46^

While fluorescence microscopy is the most commonly used method for the validation of surface gradient formation, it requires fluorophore-labeling, which can be labor intensive and may interfere with structure and activity of the bioactive gradient. An alternative and label-free method for analyzing molecular compositions directly on surfaces is matrix assisted laser desorption ionization mass spectrometry imaging (MALDI-MSI).^47^ This technique represents a unique characterization strategy that is well-established for tissue samples but is gaining more interest in the biomaterials community.^48−52^ This soft-ionization technique provides spatially resolved mass spectra of molecules with a lateral resolution of approximately 10 μm. By scanning the surface and post processing the mass spectra, images can be obtained depicting the two-dimensional intensity distribution of individual components, such as bioactive moieties or their precursor molecules. Consequently, this technique has several benefits by rapidly identifying intact chemical species on coated surfaces, in contrast to other surface detection methods like Raman or fluorescence spectroscopy.^53^ MALDI-MSI measurements for the characterization of different surface coatings rather than tissue and cell samples are scarce.^54^ Our study demonstrates the great potential of the MALDI-MSI technique for characterizing and quantifying molecular surface gradients to control cellular attachment.

**Scheme 1:**
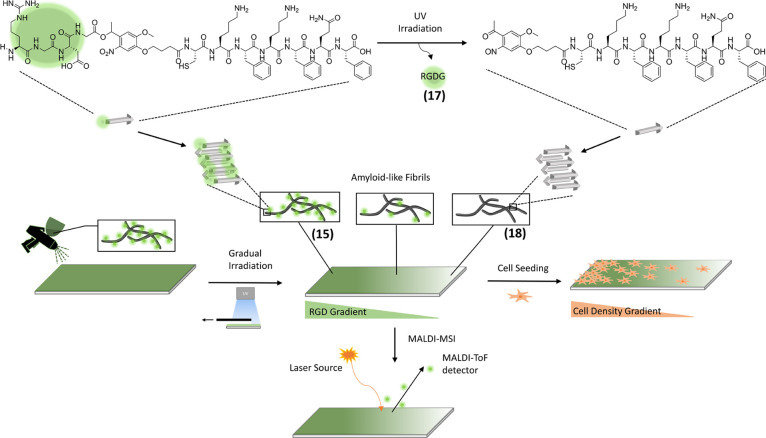
Overview of the Workflow RGD-PCL-CKFKFQF peptides (**15**) form amyloid-like fibrils. After cleavage of the RGD-moiety (**17**), the remaining peptide maintains its amyloid-like fibril morphology (**18**). Spray-coating of glass-surfaces with amyloid-fibril solution (**15**) and gradual irradiation with UV-light of the-fibril (**15**)-coated surface. Direct characterization of the gradual distribution of cleaved RGDG (**17**) and the precursor-fibril (**15**) via MALDI-MSI is feasible. A549 cell seeding on the RGD-gradient surface and incubation for 24h leads to a gradual cell distribution.

Herein, we present a straightforward method to generate substrates coated with amyloid-like nanofibrils that present gradual concentrations of bioactive epitopes to control cell adhesion with low spatial resolution. By connecting the RGD-motif via a photocleavable linker (PCL) to the bioactive self-assembling peptide CKFKFQF,^9^ a photocontrollable peptide RGD-PCL-CKFKFQF was designed. CKFKFQF nearly quantitatively (95%) assembles into amyloid-like nanofibrils in physiological environment and supports cellular adhesion and growth^9^ as well as enhanced viral transduction.^20^ The PCL-attached RGD motif is cleaved in a dose-dependent manner upon exposure to UV-light, thus generating a gradual distribution of the RGD moiety over cm-length scales. Homogenous distribution of the nanofibrils on different substrates was achieved by simple spray-coating. The molecular gradients were imaged directly, without the need for additional labels by MALDI-MSI. A two-dimensional map of the epitope-presenting substrate was achieved and cells adjusted their adhesion behavior according to the density of the RGD-epitope on the substrate (Scheme 1). This versatile platform could be employed for bio and tissue engineering, in which spatial control over cell growth can be achieved on an anisotropic distribution of bioactive molecules, mimicking the ECM in certain aspects.^55−58^

## Materials and Methods

### Materials

OymaPure, Arg(Pbf)-OH, Fmoc-Gly-OH, Fmoc-Asp(OtBu)-OH, Fmoc-Cys(Trt)-OH Fmoc-Lys(Boc)-OH, Fmoc-Phe-OH, Fmoc-Gln(Trt)-OH, and Fmoc-Phe-Wang resin were purchased from Novabiochem. *N*-Ethyldiisopropylamine (DIPEA), piperidine (≥99.5% for peptide synthesis), and trifluoroacetic acid (TFA, ≥99.9%) were obtained from Carl Roth. Dimethylformamide (DMF for peptide synthesis), diethyl ether, and dimethyl sulfoxide (DMSO, ≥99.97+%) were purchased from Acros Organics. Acetonitrile (HPLC grade) was purchased from Fisher Scientific. Syringe filters Minisart SRP (0.20 μm) were obtained from Sartorius. Glass coverslips (24 × 50 mm) were obtained from Hirschmann and glass coverslips (Ø = 13 mm) were purchased from Fisher Scientific. ITO-coated glass slides for scanning electron microscope (SEM) (15 × 20 mm) were obtained from Ossila and for MALDI-MSI (25 × 75 mm) were purchased from Bruker Daltonics. LE Agarose was obtained from Biozym Scientific. A549 cells, Dulbecco’s Modified Eagle’s Medium (DMEM, 4.5 g/L glucose/glutamine), penicillin/streptavidin, fetal bovine serum (FBS), and minimum essential medium non-essential amino acids (MEM NEAA, 100×) were purchased from Thermo Fisher Scientific. The ProteoStat Amyloid Plaque detection Kit was purchased from Enzo Life Sciences, Inc. α-Cyano-4-hydroxycinnamic acid (HCCA) was purchased from Sigma-Aldrich.

### Linker Synthesis

Photocleavable (PCL, **8**) and nonphotocleavable (NCL, **14**) linkers were synthesized according to a literature procedure (Schemes S1 and S2).^5^

### Solid-Phase Peptide Synthesis and Characterization of RGD-PCL-CKFKFQF (**15**) and RGD-NCL-CKFKFQF (**16**)

**Figure 1:**
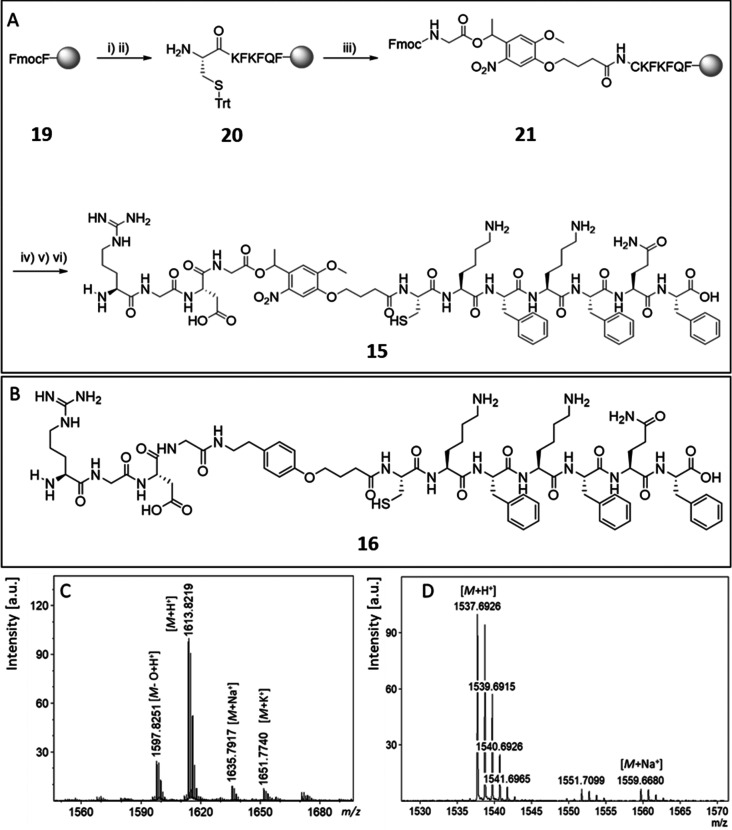
(A) Microwave assisted solid-phase peptide synthesis of the photocleavable peptide RGD-PCL-CKFKFQF (**15**): (i) Fmoc deprotection; (ii) coupling of Fmoc-Gln(Trt)-OH/Fmoc-Phe-OH/Fmoc-Lys(Boc)-OH/Fmoc-Cys(Trt)-OH; (iii) coupling of PCL; (iv) coupling of Fmoc-Asp(OtBu)-OH/Fmoc-Gly-OH/Fmoc-Arg(Pbf)-OH; (v) Fmoc deprotection; (vi) resin cleavage. (B) Nonphotocleavable peptide RGD-NCL-CKFKFQF (**16**). (C) MALDI-ToF-MS spectra of the purified peptide (**15**) confirming successful synthesis: calcd for [M + H]^+^, 1613.74 g/mol; found 1613.82 *m*/*z*, calcd for [M + Na]^+^, 1635.72 g/mol; found 1635.79 *m*/*z*., calcd for [M+K]^+^, 1651.694 g/mol found 1651.77 *m*/*z*. (D) MALDI-ToF-MS spectra of the purified peptide (**16**): calcd for [M + H]^+^, 1537.76 g/mol; found 1537.69 *m*/*z*., calcd for [M + Na]^+^, 1559.74 g/mol; found 1559.66 *m*/*z*.

Peptides were synthesized by using an automated microwave peptide synthesizer (CEM, Liberty Blue) at a 0.1 mmol scale using the Fmoc-l-Phe-Wang resin according to the standard coupling strategy (Supporting Information, section 3.3). The coupling reaction of the PCL (5 equiv, 290 mg) or NCL (5 equiv, 251 mg) to the peptide was performed manually in 1 mL of DMF with HBTU (5 equiv, 190 mg) and DIPEA (10 equiv, 175 μL) for 48 h at room temperature. The peptide was cleaved off the resin through treatment with 2 mL of TFA containing 2.5% water and 2.5% tri*iso*propylsilane (TIPS) for 2 h. This solution was added to cold diethyl ether (40 mL) and afterward centrifuged at 3000 rpm for 15 min to afford a white precipitate. The precipitate was dissolved in water and 0.1% TFA and purified via HPLC using a gradient of water and acetonitrile containing 0.1% TFA as the mobile phase. After lyophilization overnight, a white solid was obtained (47 mg; yield = 29%). The MALDI spectrum is shown in Figure 1B: theoretical [M + H]^+^= 1613.74 g/mol; found [M + H]^+^= 1613.82 g/mol.

### Photocleavage Kinetics in Solution

The kinetic study was performed using an analytical HPLC system by Shimadzu equipped with the following modules: DGU-20A5R, LC-20AT, CBM-20A, SPD-M20A, SIL-10ACHT, and CTO-20AC. In the analytical scale, the column Zorbax XDB-C18, 9.4 × 250 mm, 5 μm pore size was used. The eluent was a gradient from 5% ACN in water with 0.1% TFA to 80% ACN in 45 min. The peptide was dissolved in DMSO at a concentration of 10 mg/mL. This solution was diluted in water to a concentration of 1 mg/mL and irradiated with UV light (365 nm). 50 μL of each sample (*t*_0_ = no irradiation, *t*_1_ = 10 s, *t*_2_ = 30 s, *t*_3_ = 60 s, *t*_4_ = 5 min, *t*_5_ = 6 min, *t*_6_ = 10 min, *t*_7_ = no irradiation and incubation for 24 h) was injected.

### Nanofibril Formation

Peptides were dissolved in DMSO to obtain a 10 mg/mL stock solution, which was further diluted in Milli-Q water to 1 mg/mL. The pH value of this solution was adjusted to pH 7.4 with 0.1 M NaOH or 0.1 M HCl. The solution was incubated at room temperature for 24 h to facilitate fibril formation.^9^

### Amyloid Fibril Characterization from Solution

For TEM measurements, the nanofibril formation was performed as previously described. Carbon-film-coated copper grids were plasma-etched for 30 s at 20% intensity, before 4 μL of the preincubated peptide solution was pipetted on the grid and incubated for 5 min. Then, the solution was removed with filter paper and the grid was stained with 4% uranyl acetate solution for 2.5 min. The grids were washed three times with Milli-Q water and left to dry before measurement. TEM measurements were conducted using a Jeol 1400 electron microscope operated at 120 kV voltage and equipped with a CCD camera. ImageJ software was used for image processing. ThioflavinT (ThT) assay was performed by using nanofibril solutions that were prepare as previously described. Ten microliters (50 μM) of a ThT solution was pipetted in a black 384 well-plate and 2 μL of the nanofibril solution (1 mg/mL) were added. For reference PBS (2 μL) instead of fibril solution was added. The solutions were mixed and incubated for 15 min at room temperature to allow intercalation of ThT dye with potential cross-β-sheet structures.^16^ Subsequently, fluorescence emission was recorded λ_em_= 488 nm upon excitation at λ_ex_ = 440 nm with 10 nm bandwidth and multiple reads per well (3 × 3). Fluorescence intensity was measured using a Spark 20 M microplate reader by the company Tecan Group, Ltd. Data processing was performed with Origin software. FT-IR spectra of solid samples were recorded after lyophilization of fibril solutions using a Bruker Tensor II spectrometer equipped with a diamond crystal as ATR element with a spectral resolution of 2 cm^–1^, each spectrum was an average of 40 scans. The data was processed with Origin software.

### Characterization of Amyloid Fibril-Coated Surfaces

For SEM measurements, the ITO coated glass slides were spray-coated with an aqueous solution of preformed nanofibrils (see standard protocol) with the concentration of 0.1 mg/mL. Scanning electron measurements were performed on a Hitachi SU8000 instrument using the declaration mode with the top-detector. Various spots on the whole sample were evaluated while measuring. Unless stated otherwise the acceleration voltage was 0.1 kV. In the ProteoStat assay, 1 μL of the ProteoStat stock solution was diluted with 10 μL of assay buffer and 990 μL of Milli-Q water and pipetted on the fibril-coated surfaces. The samples were then incubated in the dark for 15 min. Images were taken with Leica DM2500 microscope coupled to a Leica DFC2000GT camera with the Rhodamine filter (fluorescence emission was recorded λ_em_ = 585/40 nm upon excitation at λ_ex_ = 546/10 nm) and processed with the software ImageJ.

### Precoating with Agarose

Glass slides, that were precleaned with *iso*propanol and Milli-Q water, were then immersed in a hot aqueous agarose solution (1 wt %) and air-dried before further usage.

### Fabrication of Nanofibril-Coated Surfaces

Nanofibrils were formed according to the standard protocol. Directly before usage, the solution was diluted to a concentration of 0.1 mg/mL solution with Milli-Q water and spray-coated with an air-brush (nozzle size 0.3 mm) on diverse surfaces. Here, 1 mL of the peptide solution was used for an agarose coated glass slide (24 × 50 mm) and 2 mL of the peptide solution for agarose coated glass slides (Ø = 13 mm) for cell tests. Two mL of the peptide solution was used for an ITO-coated glass slide for MALDI-MSI (25 × 75 mm) and 1 mL peptide solution for 4 ITO-coated glass slides for SEM measurements (15 × 20 mm).

### Atomic Force Microscopy

Atomic force microscopy was conducted in dry state with a Bruker Dimension FastScan BioTM atomic force microscope, which was operated in Tapping mode. AFM probes with a nominal force constant of 26 N/m and resonance frequency of 300 kHz (OTESPA-R3, Bruker) were used. Samples were scanned with scan rates between 0.6 and 1 Hz. Images were processed with NanoScope Analysis 1.8.

### Fabrication of RGD-Gradients

Fibril-coated surfaces were gradually irradiated (365 nm) using a programmable moving stage. (0.083 mm/s) The dried samples were placed in a distance of 2 cm to the lamp in a radiation-insulated chamber at room temperature. Irradiation of samples was conducted with LED from Opulent Americas (Starboard Luminus SST-10-UV-A130) with a peak wavelength at 365 nm and a current of 1 A and radiant flux of 875 mW. The emission spectrum of the LED was measured via an Ulbricht sphere (Figure S3).

### A549 Cell Culture

A549 cells were cultured in DMEM (4.5 g/L glucose/glutamin) supplemented with 1% penicillin/streptavidin, 10% FBS, 1% MEM NEAA. During cultivation, the medium was changed every 2–3 days. Round, 13 mm-diameter glass coverslips coated with agarose were transferred to a 6-well plate (3 glass coverslips per well), and cells were seeded at a density of 4 × 10^5^ cells/well. For the RGD-gradient, a coated glass coverslip (2.4 × 5.0 cm) was transferred to a 15 cm Petri dish, and cells were seeded at a density of 1 × 10^6^ cells/dish. After 24 h, the surfaces were washed with fresh DMEM and imaged using a Leica DM2500 microscope coupled to a Leica DFC2000GT camera. An average of three fields of view per coverslip were imaged. The number of adherent cells on the surface were analyzed by ImageJ software. All experiments were performed in biological triplicates with three technical replicates. For calceine staining 1 mL of a 1 mg/mL Calceine-AM solution was added to the medium and incubated for 30 min. The images were taken with a FITC filter (fluorescence emission was recorded λ_em_ = 527/30 nm upon excitation at λ_ex_ = 480/40 nm) and processed with ImageJ.

### Cell Viability Analysis

The cell viability against peptide nanofibrils was quantified using the CellTiter-Glo Assay (Promega G7571). Prior treatment, cells were seeded with a density of 9.000 cells/well. Peptides were preincubated via standard protocol to form fibrils and the solution was diluted with DMEM medium to create final concentrations of 0.1, 0.02, and 0.01 mg/mL. Staurosporine (1 μM) was added as negative control, while medium alone was used as positive control and were applied 24h after cell seeding, followed by incubation with cells for 24 h. The CellTiter Glo Assay was performed 48 h after cell seeding according to manufacturer’s instructions. Luminescence was detected using a GloMax Multi 96-well plate reader (Promega).

### MALDI-ToF-MSI

Relative label-free quantitation of precursor (RGD-PCL-CKFKFQF (**15**)) and fragment ions (RGDG (**17**)) were carried out by comparison of the corresponding ion signal intensities in mass spectra recorded by a MALDI-TOF mass spectrometer (rapiflex TOF/TOF, Bruker, Bremen, Germany). Acquisition of spectra were carried out in the reflection mode using the software Compass 2.0 (Bruker GmbH, Bremen, Germany) and FlexImaging 5.0, (Bruker GmbH Bremen, Germany). Utilizing a TM-Sprayer (HTX-Imaging, HTX Technologies LLC) prior to the analysis the fibril-coated and UV-irradiated surface of the ITO slide was spray-coated with a solution of MALDI matrix α-cyano-4-hydroxycinnamic acid (HCCA) (Sigma, Germany), which was in a concentration of 10 mg/mL in a solution of 70% ACN, 30% H_2_O, and 0.2% TFA. The spray method utilized was provided by the manufacturer featuring the following parameters: nozzle temperature 75 °C, nozzle height 40 mm, solvent flow 0.12 mL/min, *z*-arm velocity 1200 mm/min, N_2_ pressure 10 psi, four passes subsequently in a crisscross moving pattern, and a track spacing of 3 mm. The matrix-coated slide was introduced into the mass spectrometer by placing it into a glass slide adapter II target. An area of 60 mm × 3.5 mm was scanned in steps of 100 μm using a laser profile M5.^59^ A pixel size of 100 μm × 100 μm, 35% laser intensity, and a laser pulse repetition rate of 10 kHz was used.

To visualize the distribution of peak intensities across the measured area, we used the software FlexImaging 5.0 (Bruker, Germany). A color gradient displays the distribution of ion signal intensities^4^ normalized to total ion count (TIC) (Figure 4F, G).

### Photocleavage Kinetic on Surfaces

The cleavage kinetic was performed by using MALDI-ToF-MSI. Here, the ITO-coated glass slides were washed with Milli-Q water and *iso*propanol and dried. A fibril solution (RGD-PCL-CKFKFQF (**15**); 0.1 mg) was prepared using the standard protocol. After 24 h of incubation, these preformed fibrils were spray-coated on the ITO glass slides and irradiated for increasingly longer times (0, 1, 2, 3, 4, 5, 6, 8, and 10 min) with UV light (365 nm) by using a photomask. Since the wavelength of the UV light used for the photo cleavage experiment is very close to the one of the Nd:YAG-laser (355 nm) used for desorption/ionization in the MALDI process, it is expected that some photo cleavage can occur. In order to minimize this effect to both precursor (**15**), as well as fragment (**17**), ion yields the laser power was set to a value very close to the desorption threshold of the precursor (35%) so that no substantial ion yield of RGDG (**17**) on the nonirradiated side of the ITO glass slide was detected.

## Results and Discussion

### Design and Synthesis of a Photoresponsive Self-Assembling Peptide

To manufacture biocompatible coatings with controllable bioactivity, we chose a short peptide motif, CKFKFQF, as a supramolecular backbone.^9,20^ The fibril-forming sequence was extended at the N-terminus by a photocleavable nitrobenzyl linker (PCL) to connect bioactive epitopes to the surface of the nanofibrils. A short RGD sequence served as a model epitope, resulting in the RGD-PCL-CKFKFQF peptide (**15**). Synthesis of this molecule was achieved through Merrifield solid phase synthesis (Figure 1), and a subsequent purification was performed on a reversed phase high performance liquid chromatography (RP-HPLC, Supporting Information, section 2.2). Successful synthesis was confirmed by MALDI-ToF-MS (*m/z* = 1613.82 [M + H]^+^, *m*/*z* = 1635.79 [M + Na]^+^, *m*/*z* = 1651.77 [M + K]^+^) and LCMS (Figure S13) with a total yield of 29%. The loss of an oxygen atom of the nitrobenzyl group that emerges because of the high laser intensity during the MALDI measurement is found as well (*m*/*z* = 1597.82 [M – O + H]^+^).^60^

**Figure 2:**
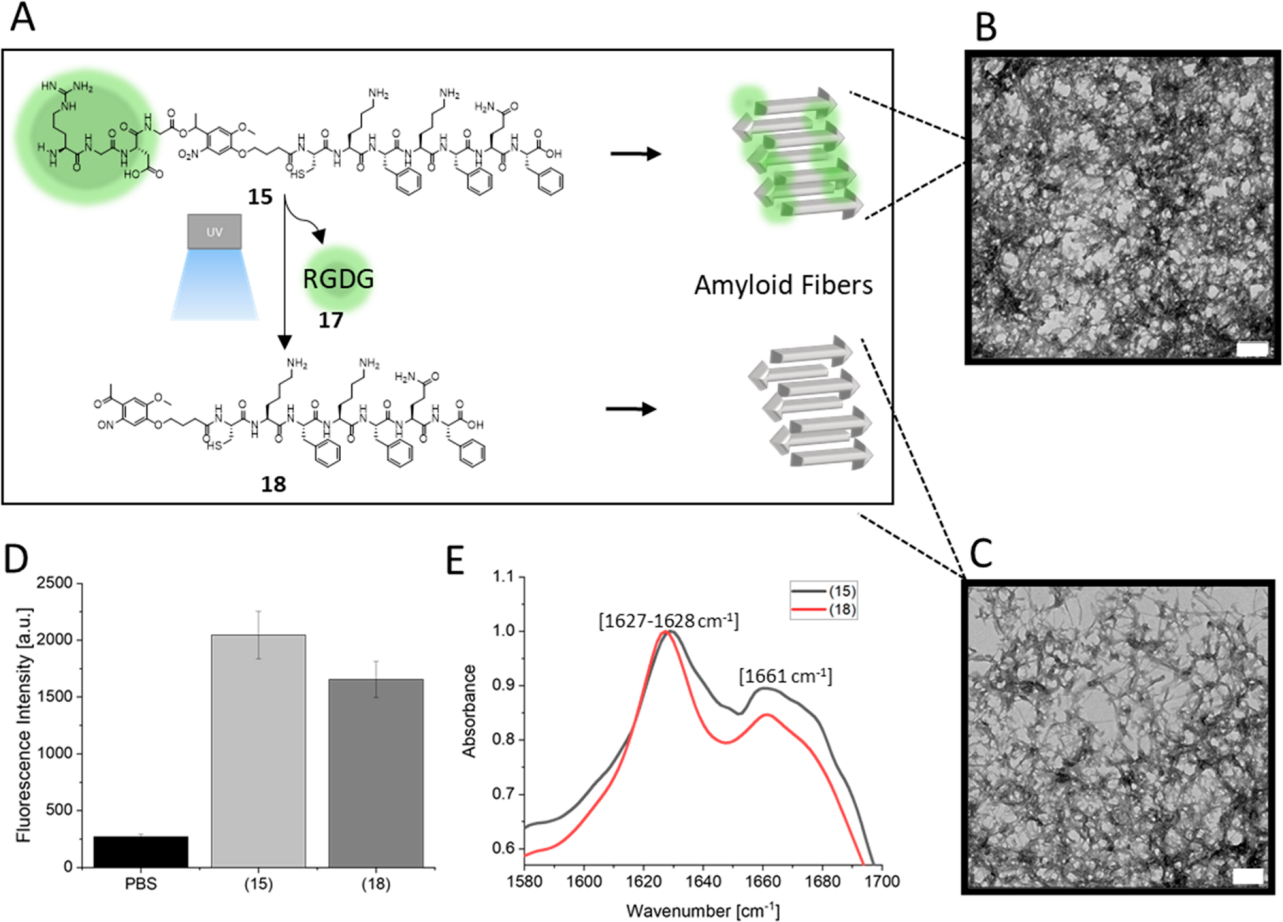
Peptide nanofibrils with photocleavable epitopes. (A) Schematic illustration of peptide nanofibril formation and UV-induced cleavage of RGDG (**17**) from peptide **15** resulting in **18**. (B) TEM images (scale bars = 0.2 μm) of peptide nanofibrils after 24 h incubation of a 1 mg/mL peptide solution in the dark. (C) Peptide nanofibrils after UV-irradiation for 10 min. (D) ThT assay shows high fluorescence for nanofibrils before and after UV irradiation. PBS served as control. (E) FT-IR spectra of peptide structures show characteristic amyloid signals for both UV treated (**18**, red line) and untreated (**15**, black line) samples.

RGD-PCL-CKFKFQF (**15**) forms amyloid nanostructures very similar as the literature-known peptide sequence CKFKFQF^9^ after incubation for 24 h in water at pH 7.4 (Figure 2B). After UV-irradiation of the preformed fibrils, the RGD epitope (**17**) is cleaved off and released (**18**) while fibril morphology is maintained (Figure 2A). To determine, whether these nanofibrils have β-sheet structures, the fibril solution was stained with thioflavin T (ThT). The so-called ThT-assay can indicate the presence of amyloid structures by an increase in fluorescence intensity of the ThT molecule after binding to the beta-sheets of amyloids.^61^ A distinct increase in the fluorescence intensity is observed for RGD-PCL-CKFKFQF (**15**) (Figure 2D), indicating that β-sheet-rich amyloid fibrils are formed. The existence of β-sheet structures was further supported by Fourier transform infrared spectroscopy (FT-IR) measurements (Figure 2E). Here, the fibril containing samples showed absorbance at 1628 and 1661 cm^–1^ that correspond to β-sheet (1628 cm^–1^ in the amyloid Aβ1–40 peptide), and β-turn structures (1662 cm^–1^ in Aβ1–40 peptide), respectively.^62^ The short peptide **15**, as well as its fragment (**18**), can rearrange to a larger structure with characteristic FT-IR absorbance similar to the amyloid structure of the Aβ1–40 peptide.

To assess whether photocleavage of the nitrobenzyl linker affects fibril formation, a freshly prepared solution of peptide **15** was UV-treated for 10 min and subsequently incubated for 24 h similar to the standard fibril formation procedure to yield the irradiated peptide **18**. The TEM measurements of **18** reveal fibrillar structures that reveal similar morphologies to those of **15** (Figure 2C). Likewise, the ThT-assay displayed an increased fluorescence intensity indicating the presence of amyloid structures for nonirradiated (**15**) and irradiated (**18**) structures (Figure 2D). Finally, FT-IR spectra of both, irradiated and nonirradiated samples, exhibit absorbance at 1627 (**18**), 1628 **(15**), and 1661 cm^–1^ (**15** and **18**) confirming that the UV irradiation does not affect the secondary structures, especially the high β-sheet content of the nanostructures (Figure 2E). The results on the assembly both peptide sequences are comparable to CKFKFQF,^9^ indicating that the presence of the photocleavable group and RGD does not interfere with CKFKFQF assembly.

**Figure 3:**
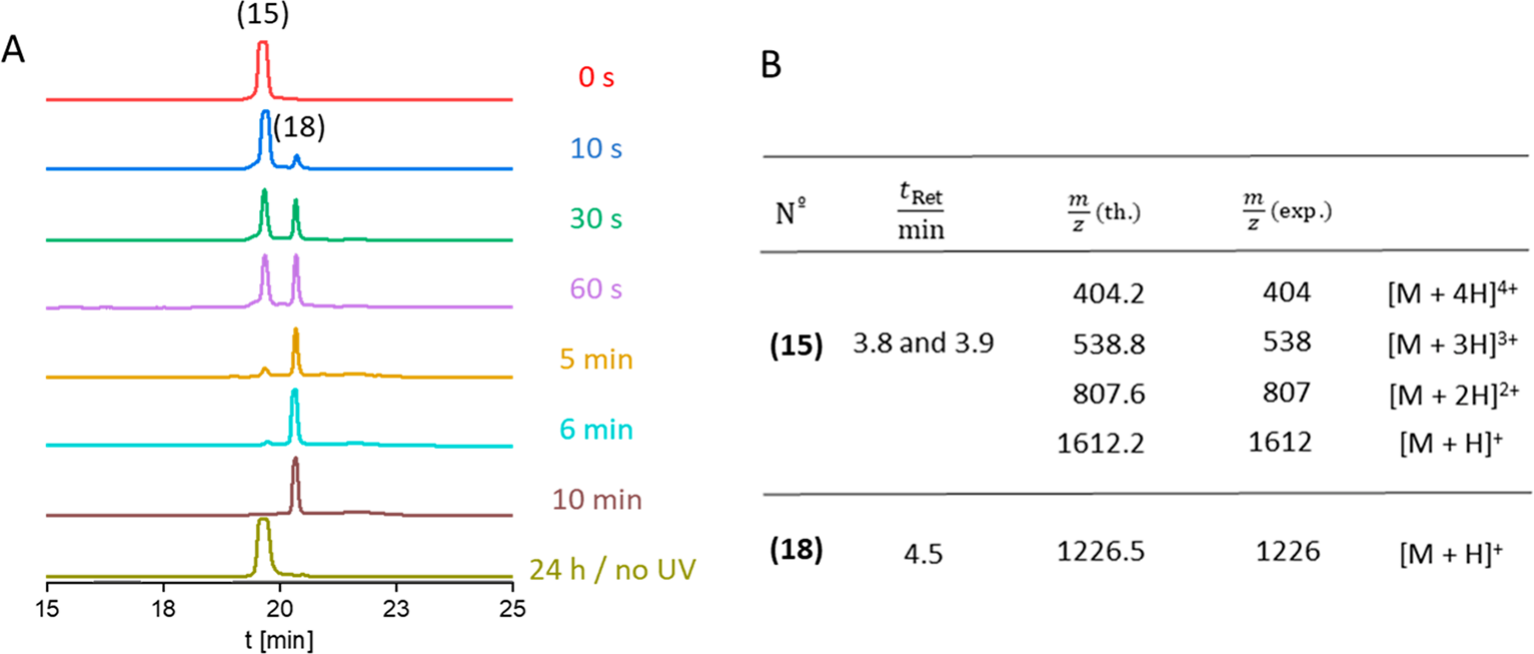
(A) HPLC spectra of peptide **15** after different UV-treatment times in aqueous solution. After 6 min of irradiation, the precursor molecule **(15)** is completely converted to **18**. (B) Assignment of signals found in LC-MS measurements of samples before and after UV treatment. *t*_Ret_ is the retention time, *m*/*z* (th.) is the theoretical molecular weight, and *m*/*z* (exp.) is the experimental molecular weight.

The photocleavage kinetics of **15** were determined in time-dependent measurements (Figure 3). Aliquots from a solution of **15** were withdrawn at intervals ranging from 0 s up to 10 min of UV-irradiation, and they were analyzed by HPLC. The signal at a retention time of 19.67 min, corresponding to the intact peptide **15**, decreases in favor of a new signal at a retention time of 20.34 min with increasing irradiation time (Figure 3A). Using LC-MS measurements (Figure 3B, Figure S15), the new signal at 20.34 min retention time was assigned to the fragment **18** with [M + H]^+^ = 1226 *m*/*z* that occurs after photocleavage. The UV-irradiation treatment rapidly cleaves the RGD motif off peptide **15** as a clear signal for **18** is visible after 10 s, and the initial peptide **15** has been almost completely consumed after 6 min (Figure 3A). The half-life of **15** under UV irradiation was determined to be *t*_1/2_ = 1.66 min (Figure S16). In the absence of light, **15** remains stable for a minimum of 24 h, as shown by HPLC (Figure 3A, beige line), confirming that the UV-irradiation triggers the cleavage reaction.

The control peptide RGD-NCL-CKFKFQF **16** containing a nonphotocleavable linker did not show any changes in HPLC elution time after UV exposure (Figures S4 and S8) (**16**) and had the same fibrous morphology and amyloid characteristics as peptide **15** (Figures S5–S7). In summary, our data suggests that peptide **15** can release the RGD motif by UV irradiation, and the degree of cleavage can be controlled by adjusting the irradiation time without affecting fibril morphology. Consequently, this peptide nanofibril platform combines stability under ambient conditions and dose-dependent UV-induced cleavage making the system suitable for generating biofunctional gradients.

### Functional Gradients on Surfaces

**Figure 4:**
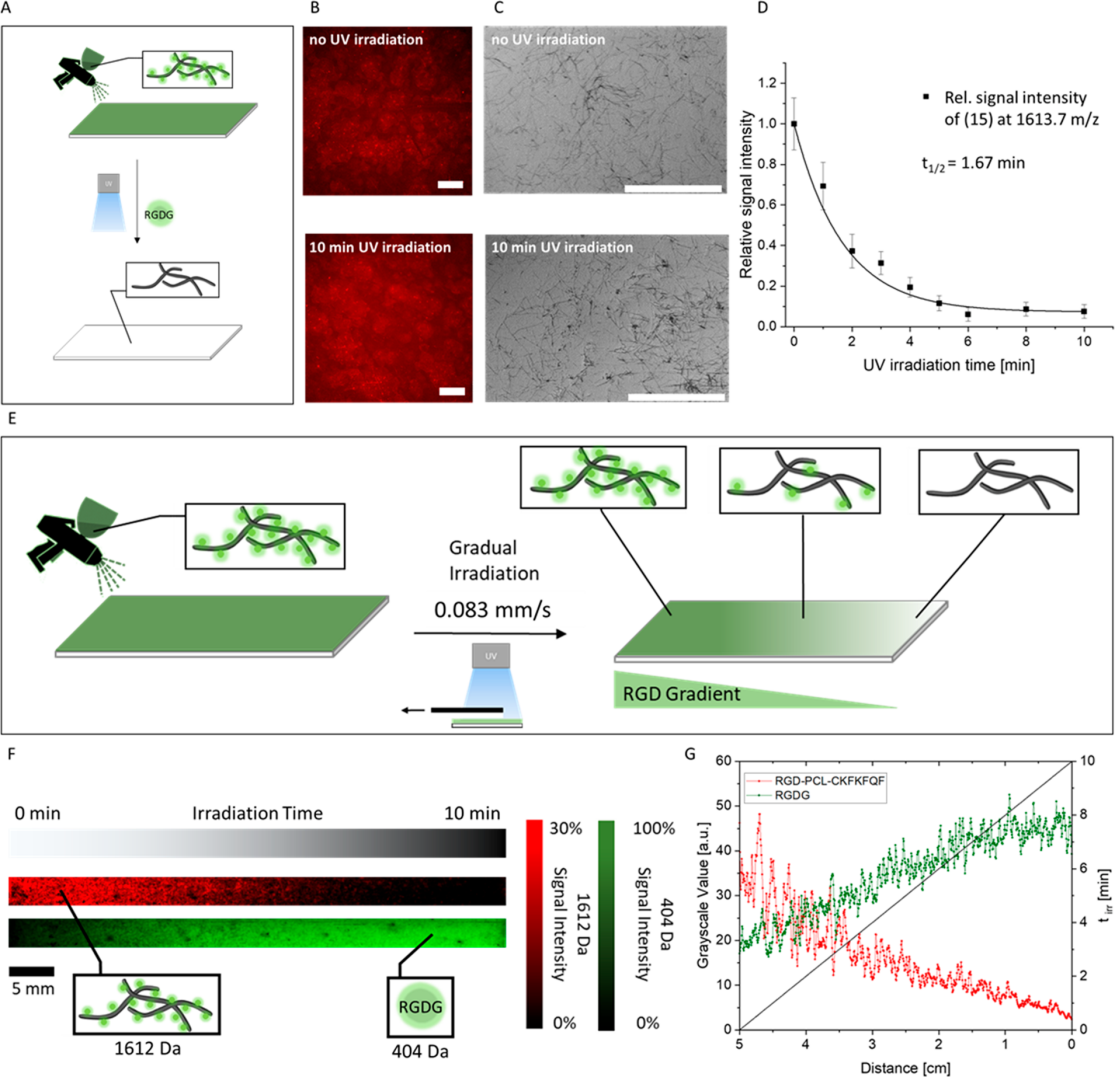
(A) Illustration of the spray-coating procedure with fibrils on surfaces and the subsequent UV-treatment of the dried sample to cleave off the bioactive RGD epitope from the fibrils. (B) Fluorescence microscopy images of ProteoStat stained, fibril coatings before (top) and after a 10 min UV treatment (bottom) (scale bar = 200 μm) show homogeneous coverage with ProteoStat active structures. (C) SEM images show the homogeneous fibril coatings before (top) and after a 10 min UV treatment (bottom) (scale bar = 5 μm). D) MALDI-ToF-MS measurement of UV-irradiation induced degradation of **15**. The sample was irradiated for 0, 1, 2, 3, 4, 5, 6, 8, and 10 min on a dried state coated on ITO-glass substrate. The MALDI signal intensities at 1613.7 *m*/*z* corresponding to RGD-PCL-CKFKFQF (**15**, black data points) were set relative to nonirradiated **15** at time 0 and fitted (black line) to first-order kinetics (*t*_1/2_ = 1.67 min). (E) Scheme of the preparation of the RGD-bound fibril gradient on the surface. (F) MALDI-MSI results show a gradual distribution of the intact RGD-fibrils (**15**, red) and the corresponding inverse gradient from the cleaved fragment RGDG (**17**, green) over a 5 cm distance. (G) The gray scale plot of MALDI-MSI results from panel B showcases an irradiation time dependent decrease of precursor signal (**15**, 1612 Da) and increase of RGDG-fragment (**17**, 404 Da).

Next, surface coatings for the preparation of cell instructive gradients were prepared (Figure 4). To accomplish surface-bound gradients of the bioactive epitope RGD, the peptide nanofibrils (**15**) were first coated on glass slides using a simple spray coating method. In principle, this method can be used to deposit bioactive fibrils on any substrate including nonflat geometries. Beside glass substrates, indium tin oxide (ITO)-coated glass slides for SEM and MALDI-MSI measurements, as well as agarose-coated glass slides, for further cell adhesion tests were spray-coated analogously. The homogeneity of the fibril coating was analyzed by fluorescence microscopy of ProteoStat Amyloid Plaque detection Kit stained substrates. The increased fluorescence signal over the entire surface confirms the presence of amyloid-like surface structures (Figure 4B) indicating that a homogeneous fibril coating from peptide **15** was achieved. These results were further supported by SEM measurements, in which the deposition of a thin layer of single fibrils is clearly visible (Figure 4C). The thickness of the coatings was determined to be 25 ± 5 nm by atomic force microscopy (Figure S17). The amyloid-like structures of the fibrils also remain after UV exposure of 10 min on the surface (Figure 4C, bottom). In summary, this spray-coating method provides a fast and easy fabrication route for homogeneous coatings of peptide fibrils on various substrates.

To evaluate the cleavage kinetics of **15** in the dry state, the peptide fibril coated ITO-glass substrates were exposed to UV light for various time durations. HCCA was applied as a matrix prior to MALDI measurements on substrates. The relative signal intensity at 1613.7 *m*/*z* of irradiated fibrils to the nonirradiated fibrils indicates the quantity of remaining precursor **15** on the surface. The results show an exponential decay with first-order kinetics and a photolysis half-life of 1.67 min (Figure 4D), which is in very good agreement to the value determined by HPLC (*t*_1/2_ = 1.66 min, Figure S16) and comparable to a literature report (*t*_1/2_ = 1.9 min) measured via a fluorescence-based approach with the same photolinker group.^5^ In addition, no further decrease in signal intensity was observed after 6 min of irradiation time, indicating that the photocleavage reaction is completed. Interestingly, a residual signal for the intact peptide **15** at about 7% relative intensity remains after 10 min of UV irradiation, which can be traced back to the previously reported photoreduction of the nitro group in the photo linker in the presence of amines. This competing reaction of the nitro groups hinders complete photocleavage reaction of self-assembled samples on substrates.^63^

### Fabrication of RGD-Gradient on Glass Substrates

A gradual distribution of the RGD-motif over a 5 cm-length scale was prepared by exposing nanofibril coated glass slides to UV-light by continuously removing a UV-impermeable cover (Figure 4). The resulting substrates were analyzed in MALDI-MSI, where signals for both the intact RGD-PCL-CKFKFQF (**15**) and the smaller RGDG (**17**) fragment, which results after photocleavage could be detected. A gradual change in the signal intensities of the corresponding *m*/*z* values were observed. Here, the intact precursor molecule (**15**, 1612 Da, illustrated in red, Figure 4F) is detected with the highest signal intensity in the areas that are nonirradiated and gradually decreases with increasing irradiation time. Vice versa, the signal intensity of the cleaved fragment RGDG (**17**, 404 Da, illustrated in green) occurs more strongly in regions of long UV irradiation (Figure 4G) and decreases with decreasing irradiation time, as expected. Plotting the grayscale values of precursor (red) and cleaved fragment (green) against the length of the irradiated sample showcases an opposite trend of decrease of precursor signal (**15**, 1612 Da) and increase of RGDG-fragment (**17**, 404 Da) with longer UV irradiation. The MALDI-MSI signal value fluctuations, especially for precursor at lower UV irradiation areas, can be explained by the setting of desorption (Figure 4G). Since the applied laser power is close to the desorption threshold of the precursor, the ion yield is very vulnerable to small fluctuations of laser power, as well as changes of the threshold of ionization itself due to contamination in the irradiated area, that is, salts, as on suppression of ionization because of the codesorbing species.

Our data shows that the cleavage reaction occurs analogue to the conditions in solution and conversion from the intact molecule (**15**) to the fragment (**17**) can be controlled simply through irradiation time with a given light intensity on surfaces. In principle, bioactive patterns could also be prepared, when an appropriate photomask or a UV projector are used.

### A549 Cell-Gradient-Formation

**Figure 5:**
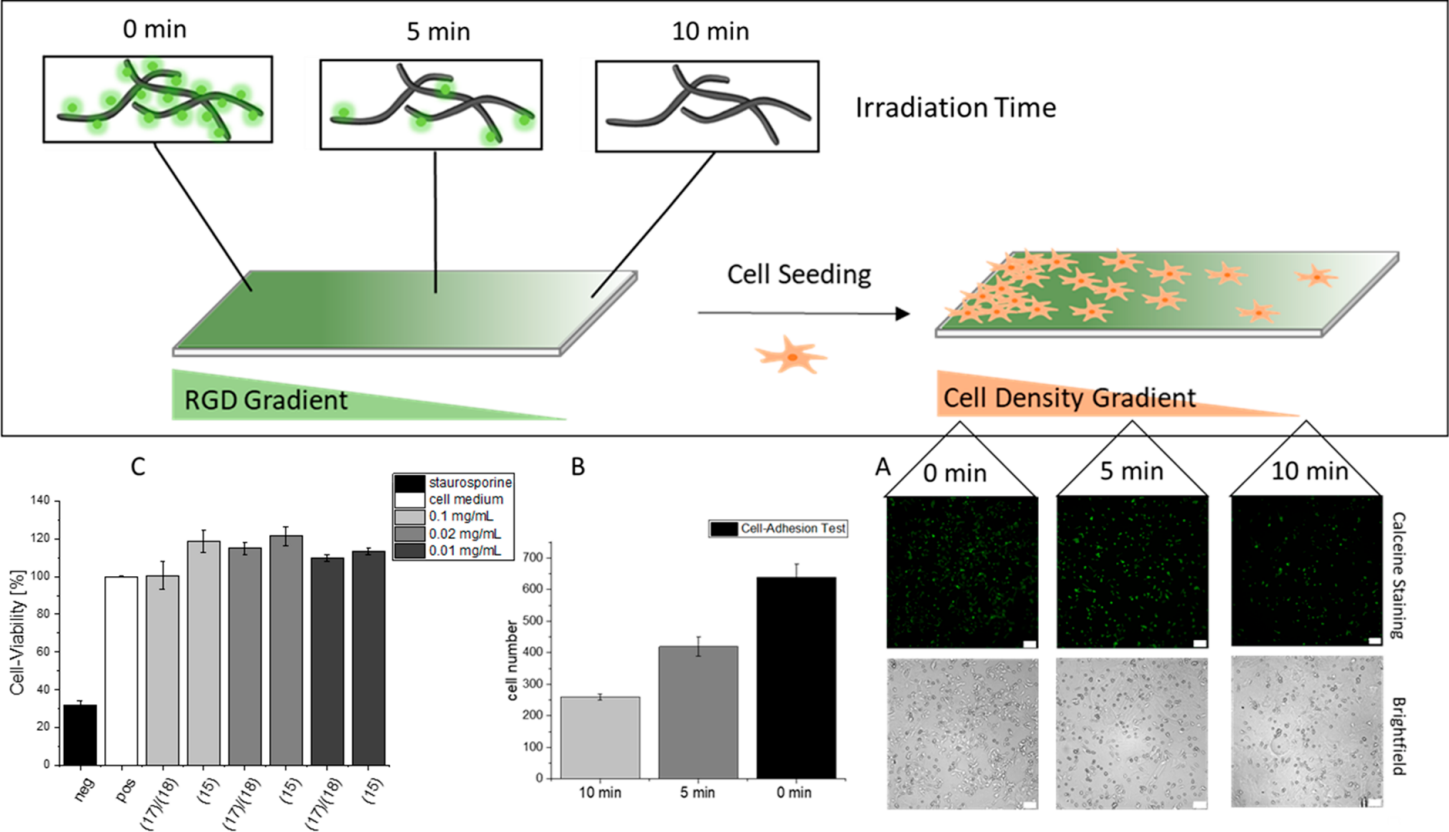
(A) Gradual irradiation of fibril coated agarose glass slide and subsequent A549 cell seeding results in a cell density gradient after incubation for 24 h. The calceine-staining shows in comparison to the brightfield images that only alive cells attach to the surface. (B) Three images of three regions (0, 5, and 10 min) were selected and the cell number was count. The result is a decrease of the cell number with an increase of irradiation. (C) The cell viability assay confirms the non toxic character of the used fibrils with **17**/**18** with irradiation for 10 min and of **15** without irradiation. Negative control is a toxic staurosporine solution and positive control are cells without added peptides (scale bar: 100 μm).

The biological response to the RGD-gradients was tested in cell culture (Figure 5). To this end, a cell-repellent precoating with agarose was applied on glass slides prior to fibril coating and irradiation, to avoid unwanted cellular adhesion of A549 cells (Figure S14). These surfaces were subsequently spray-coated with nanofibril solution and gradually irradiated as previously described, thus creating an RGD-gradient. After incubation with a suspension of A549-cells for 24 h, the substrates were analyzed for cell-attachment in three distinct regions (low, medium, and high UV irradiation) using fluorescence microscopy. Attached and alive cells were stained with calceine (Figure 5A). A significant difference in cell density is visible when comparing the three regions of the substrate (Figure 5B). As expected, the sections exposed to longer UV irradiation time show less cell attachment than those with shorter exposure times, indicating that a bioactive gradient is achieved that cells can respond to. Noteworthy, some cell attachment and spreading are observed also for fully photocleaved peptides at 100% UV irradiation site (Figures 5B and S14). This observation is in accordance with previous reports on the backbone peptide fibril CKFKFQF stimulating cell adhesion^9^ and further emphasizes the maintenance of the amyloid-like morphology after UV irradiation. A cell-viability assay confirmed the nontoxic character of both the non-UV-treated and UV-treated fibrils (Figure 5C). As a control, surfaces with the noncleavable peptide **16** were exposed to UV irradiation and subsequently seeded with A549 cells. As expected, no change in the adhesion behavior of the cells on irradiated and nonirradiated samples were detected (Figure S11). The cell-viability assay was likewise carried out and shows no toxic behavior of peptide **16** before and after UV irradiation (Figure S12). The cell adhesion results and the cell viability data confirming negligible toxicity, demonstrate that the designed amyloid-like fibrils are a promising scaffold for controlling epitope presentation and displaying cell density gradients.

## Conclusions

In summary, we developed an easy and fast strategy to create bioactive spatial gradients by spray-coating UV-sensitive, epitope functionalized amyloid-like peptide fibrils on glass slides. The bottom-up approach of the designed photocleavable peptide **15** requires the usage of a nitrobenzyl linker that allows the RGD-epitope cleavage of fibrils after the coating procedure. The cleavage was performed by irradiating the samples with 365 nm UV light. Gradients of irradiated photocleavable peptide **15** on ITO-coated glass slides were visualized by MALDI-MSI measurements, which is a facile method for precise and direct mass characterization of 2D samples covering multiple length scales (micrometers–centimeters). Exemplarily, A549 cell density gradients could be achieved by spray-coating peptide **15** on agarose coated glass slides. In addition, the amyloid fibril coatings before and after UV irradiation were nontoxic when tested in vitro. The amyloid-like fibrils show a high tendency for cell adhesion and spreading. In areas of high RGD concentrations, the number of attached cells is further enhanced 3-fold compared to areas of low RGD concentration, enabling a precise control over cell density through the underlying coating. By replacing the RGD epitope with other motifs like bioactive peptide sequences, DNA aptamers, nano- or even antibodies, different cells and their responses may be studied in regard to the gradual distribution of the respective signal. For incorporating larger epitopes, optimized self-assembling peptide sequences as well as other bioconjugation strategies, such as a postassembly conjugation, that is, strain-promoted cycloaddition reactions, may become necessary, to ensure correct spatial presentation necessary for biological function. We envision that our strategy can be used for tissue engineering in regenerative medicine, especially considering peptide nanofibrils as ECM mimicking materials.

## Supporting Information Available

Materials for linker synthesis; instrumentation details for characterization (NMR, HPLC, LC-MS, MALDI-ToF-MS, microwave reactor); synthesis of PCL (**8**); synthesis of NCL (**14**); irradiation setup; characterization of noncleavable peptide (**16**) before and after irradiation by LC-MS, TEM, ThT-assay, FT-IR, HPLC, SEM, ProteoStat assay, cell adhesion test, cell viability test; and characterization of (**15**) by LC-MS and cell adhesion test (PDF)

## References

[ref1] Lee S.; Trinh T. H. T.; Yoo M.; Shin J.; Lee H.; Kim J.; Hwang E.; Lim Y.; Ryou C. Self-Assembling Peptides and Their Application in the Treatment of Diseases. Int. J. Mol. Sci. 2019, 20 (23), 5850 10.3390/ijms20235850. PMC6928719 31766475

[ref2] Sato K.; Hendricks M. P.; Palmer L. C.; Stupp S. I. Peptide Supramolecular Materials for Therapeutics. Chem. Soc. Rev. 2018, 47 (20), 7539–7551. 10.1039/C7CS00735C. 30187042 PMC6188840

[ref3] Li Y.; Xiao Y.; Liu C. The Horizon of Materiobiology: A Perspective on Material-Guided Cell Behaviors and Tissue Engineering. Chem. Rev. 2017, 117 (5), 4376–4421. 10.1021/acs.chemrev.6b00654. 28221776

[ref4] Wu S.; Du W.; Duan Y.; Zhang D.; Liu Y.; Wu B.; Zou X.; Ouyang H.; Gao C. Regulating the Migration of Smooth Muscle Cells by a Vertically Distributed Poly(2-Hydroxyethyl Methacrylate) Gradient on Polymer Brushes Covalently Immobilized with RGD Peptides. Acta Biomater. 2018, 75, 75–92. 10.1016/j.actbio.2018.05.046. 29857130

[ref5] Sur S.; Matson J. B.; Webber M. J.; Newcomb C. J.; Stupp S. I. Photodynamic Control of Bioactivity in a Nanofiber Matrix. ACS Nano 2012, 6 (12), 10776–10785. 10.1021/nn304101x. 23153342 PMC3528833

[ref6] Wheeldon I.; Farhadi A.; Bick A. G.; Jabbari E.; Khademhosseini A. Nanoscale Tissue Engineering: Spatial Control over Cell-Materials Interactions. Nanotechnology 2011, 22 (21), 212001 10.1088/0957-4484/22/21/212001. 21451238 PMC3155808

[ref7] Chung T. W.; Liu D. Z.; Wang S. Y.; Wang S. S. Enhancement of the Growth of Human Endothelial Cells by Surface Roughness at Nanometer Scale. Biomaterials 2003, 24 (25), 4655–4661. 10.1016/S0142-9612(03)00361-2. 12951008

[ref8] Frantz C.; Stewart K. M.; Weaver V. M. The Extracellular Matrix at a Glance. J. Cell Sci. 2010, 123 (24), 4195–4200. 10.1242/jcs.023820. 21123617 PMC2995612

[ref9] Schilling C.; Mack T.; Lickfett S.; Sieste S.; Ruggeri F. S.; Sneideris T.; Dutta A.; Bereau T.; Naraghi R.; Sinske D.; Knowles T. P. J.; Synatschke C. V.; Weil T.; Knöll B. Sequence-Optimized Peptide Nanofibers as Growth Stimulators for Regeneration of Peripheral Neurons. Adv. Funct. Mater. 2019, 29 (24), 1809112 10.1002/adfm.201809112.

[ref10] Chen X.; Su Y. D.; Ajeti V.; Chen S. J.; Campagnola P. J. Cell Adhesion on Micro-Structured Fibronectin Gradients Fabricated by Multiphoton Excited Photochemistry. Cell. Mol. Bioeng. 2012, 5 (3), 307–319. 10.1007/s12195-012-0237-8. 23710258 PMC3662366

[ref11] Adamcik J.; Ruggeri F. S.; Berryman J. T.; Zhang A.; Knowles T. P. J.; Mezzenga R. Evolution of Conformation, Nanomechanics, and Infrared Nanospectroscopy of Single Amyloid Fibrils Converting into Microcrystals. Adv. Sci. 2021, 8 (2), 2002182 10.1002/advs.202002182. PMC7816722 33511004

[ref12] Glenner G. G.; Wong C. W. Alzheimer’s Disease and Down’s Syndrome: Sharing of a Unique Cerebrovascular Amyloid Fibril Protein. Biochem. Biophys. Res. Commun. 1984, 122 (3), 1131–1135. 10.1016/0006-291X(84)91209-9. 6236805

[ref13] Collinge J. Mammalian Prions and Their Wider Relevance in Neurodegenerative Diseases. Nature 2016, 539 (7628), 217–226. 10.1038/nature20415. 27830781

[ref14] Cherny I.; Gazit E. Amyloids: Not Only Pathological Agents but Also Ordered Nanomaterials. Angew. Chem., Int. Ed. 2008, 47 (22), 4062–4069. 10.1002/anie.200703133. 18412209

[ref15] Wei G.; Su Z.; Reynolds N. P.; Arosio P.; Hamley I. W.; Gazit E.; Mezzenga R. Self-Assembling Peptide and Protein Amyloids: From Structure to Tailored Function in Nanotechnology. Chem. Soc. Rev. 2017, 46 (15), 4661–4708. 10.1039/C6CS00542J. 28530745 PMC6364806

[ref16] Gačanin J.; Hedrich J.; Sieste S.; Glaßer G.; Lieberwirth I.; Schilling C.; Fischer S.; Barth H.; Knöll B.; Synatschke C. V.; Weil T. Autonomous Ultrafast Self-Healing Hydrogels by PH-Responsive Functional Nanofiber Gelators as Cell Matrices. Adv. Mater. 2019, 31 (2), 1805044 10.1002/adma.201805044. 30411838

[ref17] Pilkington S. M.; Roberts S. J.; Meade S. J.; Gerrard J. A. Amyloid Fibrils as a Nanoscaffold for Enzyme Immobilization. Biotechnol. Prog. 2009, 26 (1), 93–100. 10.1002/btpr.309. 19918761

[ref18] Reynolds N. P. Amyloid-like Peptide Nanofibrils as Scaffolds for Tissue Engineering: Progress and Challenges (Review). Biointerphases 2019, 14 (4), 040801 10.1116/1.5098332. 31284721

[ref19] Kaygisiz K.; Synatschke C. V. Materials Promoting Viral Gene Delivery. Biomater. Sci. 2020, 8 (22), 6113–6156. 10.1039/D0BM01367F. 33025967

[ref20] Sieste S.; Mack T.; Lump E.; Hayn M.; Schütz D.; Röcker A.; Meier C.; Kaygisiz K.; Kirchhoff F.; Knowles T. P. J.; Ruggeri F. S.; Synatschke C. V.; Münch J.; Weil T. Supramolecular Peptide Nanofibrils with Optimized Sequences and Molecular Structures for Efficient Retroviral Transduction. Adv. Funct. Mater. 2021, 31, 2009382 10.1002/adfm.202009382.

[ref21] Romero D.; Aguilar C.; Losick R.; Kolter R. Amyloid Fibers Provide Structural Integrity to Bacillus Subtilis Biofilms. Proc. Natl. Acad. Sci. U. S. A. 2010, 107 (5), 2230–2234. 10.1073/pnas.0910560107. 20080671 PMC2836674

[ref22] Ke P. C.; Zhou R.; Serpell L. C.; Riek R.; Knowles T. P. J.; Lashuel H. A.; Gazit E.; Hamley I. W.; Davis T. P.; Fändrich M.; Otzen D. E.; Chapman M. R.; Dobson C. M.; Eisenberg D. S.; Mezzenga R. Half a Century of Amyloids: Past, Present and Future. Chem. Soc. Rev. 2020, 49 (15), 5473–5509. 10.1039/C9CS00199A. 32632432 PMC7445747

[ref23] Jamous S.; Comba A.; Lowenstein P. R.; Motsch S. Self-Organization in Brain Tumors: How Cell Morphology and Cell Density Influence Glioma Pattern Formation. PLoS Comput. Biol. 2020, 16 (5), e1007611 10.1371/journal.pcbi.1007611. 32379821 PMC7244185

[ref24] Gras S. L.; Tickler A. K.; Squires A. M.; Devlin G. L.; Horton M. A.; Dobson C. M.; MacPhee C. E. Functionalised Amyloid Fibrils for Roles in Cell Adhesion. Biomaterials 2008, 29 (11), 1553–1562. 10.1016/j.biomaterials.2007.11.028. 18164758

[ref25] Gehlen D. B.; De Lencastre Novaes L. C.; Long W.; Ruff A. J.; Jakob F.; Haraszti T.; Chandorkar Y.; Yang L.; Van Rijn P.; Schwaneberg U.; De Laporte L. Rapid and Robust Coating Method to Render Polydimethylsiloxane Surfaces Cell-Adhesive. ACS Appl. Mater. Interfaces 2019, 11 (44), 41091–41099. 10.1021/acsami.9b16025. 31600051

[ref26] Reynolds N. P.; Charnley M.; Bongiovanni M. N.; Hartley P. G.; Gras S. L. Biomimetic Topography and Chemistry Control Cell Attachment to Amyloid Fibrils. Biomacromolecules 2015, 16 (5), 1556–1565. 10.1021/acs.biomac.5b00114. 25871317

[ref27] Bongiovanni M. N.; Scanlon D. B.; Gras S. L. Functional Fibrils Derived from the Peptide TTR1-CycloRGDfK That Target Cell Adhesion and Spreading. Biomaterials 2011, 32 (26), 6099–6110. 10.1016/j.biomaterials.2011.05.021. 21636126

[ref28] Bongiovanni M. N.; Gras S. L. Bioactive TTR105–115-Based Amyloid Fibrils Reduce the Viability of Mammalian Cells. Biomaterials 2015, 46, 105–116. 10.1016/j.biomaterials.2014.12.039. 25678120

[ref29] Ohga Y.; Katagiri F.; Takeyama K.; Hozumi K.; Kikkawa Y.; Nishi N.; Nomizu M. Design and Activity of Multifunctional Fibrils Using Receptor-Specific Small Peptides. Biomaterials 2009, 30 (35), 6731–6738. 10.1016/j.biomaterials.2009.08.044. 19765823

[ref30] Deidda G.; Jonnalagadda S. V. R.; Spies J. W.; Ranella A.; Mossou E.; Forsyth V. T.; Mitchell E. P.; Bowler M. W.; Tamamis P.; Mitraki A. Self-Assembled Amyloid Peptides with Arg-Gly-Asp (RGD) Motifs As Scaffolds for Tissue Engineering. ACS Biomater. Sci. Eng. 2017, 3 (7), 1404–1416. 10.1021/acsbiomaterials.6b00570. 33429698

[ref31] King P. J. S.; Giovanna Lizio M.; Booth A.; Collins R. F.; Gough J. E.; Miller A. F.; Webb S. J. A Modular Self-Assembly Approach to Functionalised β-Sheet Peptide Hydrogel Biomaterials. Soft Matter 2016, 12 (6), 1915–1923. 10.1039/C5SM02039E. 26702608

[ref32] Ricoult S. G.; Kennedy T. E.; Juncker D. Substrate-Bound Protein Gradients to Study Haptotaxis. Front. Bioeng. Biotechnol. 2015, 3, 40 10.3389/fbioe.2015.00040. 25870855 PMC4378366

[ref33] Roy J.; Mazzaferri J.; Filep J. G.; Costantino S. A Haptotaxis Assay for Neutrophils Using Optical Patterning and a High-Content Approach. Sci. Rep. 2017, 7 (1), 2869 10.1038/s41598-017-02993-6. 28588217 PMC5460230

[ref34] Doyle A. D.; Petrie R. J.; Kutys M. L.; Yamada K. M. Dimensions in Cell Migration. Curr. Opin. Cell Biol. 2013, 25 (5), 642–649. 10.1016/j.ceb.2013.06.004. 23850350 PMC3758466

[ref35] Benetti E. M.; Gunnewiek M. K.; Van Blitterswijk C. A.; Julius Vancso G.; Moroni L. Mimicking Natural Cell Environments: Design, Fabrication and Application of Bio-Chemical Gradients on Polymeric Biomaterial Substrates. J. Mater. Chem. B 2016, 4 (24), 4244–4257. 10.1039/C6TB00947F. 32263405

[ref36] Inagi S. Fabrication of Gradient Polymer Surfaces Using Bipolar Electrochemistry. Polym. J. 2016, 48 (1), 39–44. 10.1038/pj.2015.73.

[ref37] Chatterjee K.; Lin-Gibson S.; Wallace W. E.; Parekh S. H.; Lee Y. J.; Cicerone M. T.; Young M. F.; Simon C. G. The Effect of 3D Hydrogel Scaffold Modulus on Osteoblast Differentiation and Mineralization Revealed by Combinatorial Screening. Biomaterials 2010, 31 (19), 5051–5062. 10.1016/j.biomaterials.2010.03.024. 20378163 PMC3125577

[ref38] Norris S. C. P.; Tseng P.; Kasko A. M. Direct Gradient Photolithography of Photodegradable Hydrogels with Patterned Stiffness Control with Submicrometer Resolution. ACS Biomater. Sci. Eng. 2016, 2 (8), 1309–1318. 10.1021/acsbiomaterials.6b00237. 33434984

[ref39] Lee J.; Ku K. H.; Kim J.; Lee Y. J.; Jang S. G.; Kim B. J. Light-Responsive, Shape-Switchable Block Copolymer Particles. J. Am. Chem. Soc. 2019, 141 (38), 15348–15355. 10.1021/jacs.9b07755. 31433168

[ref40] Marklein R. A.; Burdick J. A. Spatially Controlled Hydrogel Mechanics to Modulate Stem Cell Interactions. Soft Matter 2010, 6 (1), 136–143. 10.1039/B916933D.

[ref41] Gupta M. K.; Balikov D. A.; Lee Y.; Ko E.; Yu C.; Chun Y. W.; Sawyer D. B.; Kim W. S.; Sung H. J. Gradient Release of Cardiac Morphogens by Photo-Responsive Polymer Micelles for Gradient-Mediated Variation of Embryoid Body Differentiation. J. Mater. Chem. B 2017, 5 (26), 5206–5217. 10.1039/C7TB00880E. 32264105

[ref42] Wirkner M.; Alonso J. M.; Maus V.; Salierno M.; Lee T. T.; García A. J.; del Campo A. Triggered Cell Release from Materials Using Bioadhesive Photocleavable Linkers. Adv. Mater. 2011, 23 (34), 3907–3910. 10.1002/adma.201100925. 21618293

[ref43] Ricken J.; Medda R.; Wegner S. V. Photo-ECM: A Blue Light Photoswitchable Synthetic Extracellular Matrix Protein for Reversible Control over Cell–Matrix Adhesion. Adv. Biosyst. 2019, 3 (3), 1800302 10.1002/adbi.201800302. 32627396

[ref44] Wegner S. V.; Sentürk O. I.; Spatz J. P. Photocleavable Linker for the Patterning of Bioactive Molecules. Sci. Rep. 2016, 5 (1), 18309 10.1038/srep18309. PMC4680943 26670693

[ref45] Cui J.; Miguel V. S.; Del Campo A. Light-Triggered Multifunctionality at Surfaces Mediated by Photolabile Protecting Groups. Macromol. Rapid Commun. 2013, 34 (4), 310–329. 10.1002/marc.201200634. 23225073

[ref46] Luo W.; Yousaf M. N. Tissue Morphing Control on Dynamic Gradient Surfaces. J. Am. Chem. Soc. 2011, 133 (28), 10780–10783. 10.1021/ja204893w. 21707041

[ref47] Schulz S.; Becker M.; Groseclose M. R.; Schadt S.; Hopf C. Advanced MALDI Mass Spectrometry Imaging in Pharmaceutical Research and Drug Development. Curr. Opin. Biotechnol. 2019, 55, 51–59. 10.1016/j.copbio.2018.08.003. 30153614

[ref48] Paine M. R. L.; Kooijman P. C.; Fisher G. L.; Heeren R. M. A.; Fernández F. M.; Ellis S. R. Visualizing Molecular Distributions for Biomaterials Applications with Mass Spectrometry Imaging: A Review. J. Mater. Chem. B 2017, 5 (36), 7444–7460. 10.1039/C7TB01100H. 32264222

[ref49] Medini K.; West B.; Williams D. E.; Brimble M. A.; Gerrard J. A. MALDI-Imaging Enables Direct Observation of Kinetic and Thermodynamic Products of Mixed Peptide Fiber Assembly. Chem. Commun. 2017, 53 (10), 1715–1718. 10.1039/C6CC10146A. 28102381

[ref50] Touve M. A.; Carlini A. S.; Gianneschi N. C. Self-Assembling Peptides Imaged by Correlated Liquid Cell Transmission Electron Microscopy and MALDI-Imaging Mass Spectrometry. Nat. Commun. 2019, 10 (1), 4837 10.1038/s41467-019-12660-1. 31645558 PMC6811541

[ref51] Hall-Andersen J.; Kaasgaard S. G.; Janfelt C. MALDI Imaging of Enzymatic Degradation of Glycerides by Lipase on Textile Surface. Chem. Phys. Lipids 2018, 211, 100–106. 10.1016/j.chemphyslip.2017.11.004. 29122612

[ref52] Anderson D. M.; Nye-Wood M. G.; Rose K. L.; Donaldson P. J.; Grey A. C.; Schey K. L. MALDI Imaging Mass Spectrometry of β- and γ-Crystallins in the Ocular Lens. J. Mass Spectrom. 2020, 55 (4), e4473 10.1002/jms.4473. 31713937 PMC8184062

[ref53] Li C.; Armstrong J. P.; Pence I. J.; Kit-Anan W.; Puetzer J. L.; Correia Carreira S.; Moore A. C.; Stevens M. M. Glycosylated Superparamagnetic Nanoparticle Gradients for Osteochondral Tissue Engineering. Biomaterials 2018, 176, 24–33. 10.1016/j.biomaterials.2018.05.029. 29852377 PMC6018621

[ref54] Lee J.; Choi I.; Yeo W.-S. Preparation of Gradient Surfaces by Using a Simple Chemical Reaction and Investigation of Cell Adhesion on a Two-Component Gradient. Chem. - Eur. J. 2013, 19 (18), 5609–5616. 10.1002/chem.201203215. 23463672

[ref55] Kuzucu M.; Vera G.; Beaumont M.; Fischer S.; Wei P.; Shastri V. P.; Forget A. Extrusion-Based 3D Bioprinting of Gradients of Stiffness, Cell Density, and Immobilized Peptide Using Thermogelling Hydrogels. ACS Biomater. Sci. Eng. 2021, 7 (6), 2192–2197. 10.1021/acsbiomaterials.1c00183. 33970597 PMC8207502

[ref56] Keenan T. M.; Folch A. Biomolecular Gradients in Cell Culture Systems. Lab Chip 2008, 8 (1), 34–57. 10.1039/B711887B. 18094760 PMC3848882

[ref57] Zonderland J.; Rezzola S.; Wieringa P.; Moroni L. Fiber Diameter, Porosity and Functional Group Gradients in Electrospun Scaffolds. Biomed. Mater. 2020, 15 (4), 045020 10.1088/1748-605X/ab7b3c. 32109896

[ref58] Khorshidi S.; Karkhaneh A. A Review on Gradient Hydrogel/Fiber Scaffolds for Osteochondral Regeneration. J. Tissue Eng. Regener. Med. 2018, 12 (4), e1974–e1990. 10.1002/term.2628. 29243352

[ref59] Holle A.; Haase A.; Kayser M.; Höhndorf J. Optimizing UV Laser Focus Profiles for Improved MALDI Performance. J. Mass Spectrom. 2006, 41 (6), 705–716. 10.1002/jms.1041. 16718638

[ref60] Zhan X.; Desiderio D. M. MALDI-Induced Fragmentation of Leucine Enkephalin, Nitro-Tyr-Leucine Enkaphalin, and D5-Phe-Nitro-Tyr-Leucine Enkephalin. Int. J. Mass Spectrom. 2009, 287 (1–3), 77–86. 10.1016/j.ijms.2008.08.020. 20161518 PMC2799299

[ref61] Amdursky N.; Erez Y.; Huppert D. Molecular Rotors: What Lies behind the High Sensitivity of the Thioflavin-T Fluorescent Marker. Acc. Chem. Res. 2012, 45 (9), 1548–1557. 10.1021/ar300053p. 22738376

[ref62] Adochitei A.; Drochioiu G. Rapid Characterization of Peptide Secondary Structure by FT-IR Spectroscopy. Rev. Roum. Chim. 2011, 56 (8), 783–791.

[ref63] Critchley K.; Zhang L.; Fukushima H.; Ishida M.; Shimoda T.; Bushby R. J.; Evans S. D. Soft-UV Photolithography Using Self-Assembled Monolayers. J. Phys. Chem. B 2006, 110 (34), 17167–17174. 10.1021/jp0630370. 16928013

