# Supplementary material for: Cell-Instructive Surface Gradients of Photoresponsive Amyloid-like Fibrils
Source: ACS Biomater Sci Eng. 2021 Sep 13;7(10):4798–808. doi: 10.1021/acsbiomaterials.1c00889 (PMC8512672; doi:10.1021/acsbiomaterials.1c00889)
Supplement: Supplementary file 1 — ab1c00889_si_001.pdf [file ab1c00889_si_001.pdf]

Supporting Information

Cell-Instructive Surface Gradients of Photoresponsive Amyloid-like Fibrils

Adriana Maria Ender[1#], Kübra Kaygisiz[1#], Hans-Joachim Räder[1], Franz J. Mayer[1], Christopher V. Synatschke[1], Tanja Weil[1]**

1 Department Synthesis of Macromolecules, Max Planck Institute for Polymer Research, Ackermannweg 10, 55128 Mainz, Germany

# denotes equal contribution

Correspondence to be addressed to: synatschke@mpip-mainz.mpg.de; weil@mpip-mainz.mpg.de

26 Pages, Figures S1-S17, Scheme S1, S2

Table of Content

1. Materials for Linker Synthesis 3
2. Instruments 3
   2.1. Nuclear Magnetic Resonance Spectroscopy (NMR) 3
   2.2. High-Performance Liquid Chromatography (HPLC) 3
   2.3. Liquid Chromatography - Mass Spectrometry (LC-MS) 4
   2.4. Matrix-Assisted Laser Desorption/Ionization - Time of Flight Mass Spectrometry (MALDI-ToF MS) 4
   2.5. Matrix-Assisted Laser Desorption/Ionization - Time of Flight Mass Spectrometry Imaging (MALDI-MSI) 5
   2.6. Microwave Peptide Synthesizer 5
3. Synthesis 6
   3.1. Synthesis of Photo-cleavable Linker, PCL **(8)**, according to Sur et al.[1] 6
   3.2. Synthesis of non- photo cleavable linker NCL **(14)**, according to Sur et al.[1] 13
   3.3. Standard Peptide Synthesis Strategy 17
   3.4. Irradiation 18
4. Additional Characterization RGD-NCL-CKFKFQF **(16)** 19
   4.1. LC-MS 19
   4.2. TEM 19
   4.3. ThT-Assay 20
   4.4. Fourier-Transform Infrared Spectroscopy (FT-IR) 21
   4.5. HPLC study 21
   4.6. SEM 22
   4.7. ProteoStat-Assay on Surface 22
   4.8. A549 Cell Adhesion Assay 23
   4.9. Cell-Viability Assay 23
5. Additional Characterization RGD-PCL-CKFKFQF **(15)** 24
   5.1. LC-MS 24
   5.2. A549 Cell Adhesion Assay without Gradient formation 24
   5.3. LC-MS Measurements of Non-irradiated Molecule **(15)** and Irradiated Molecule **(18)** 25
   5.4. Comparison of cleavage kinetics of **(15)** 25
   5.5. Scratch assay of ITO slides coated with (**15**) by atomic force microscopy 26
6. References 26

1. Materials for Linker Synthesis

Dimethylformamide (DMF, for peptide synthesis), dimethylsulfoxide (DMSO) and 4-dimethylaminopyridine (DMAP) were purchased from Acros Organics. EDC hydrochloride, tyramine hydrochloride, 3,4-dihydro-2H pyrane, pyridinium *p*-toluenesulfonate, potassium iodide (KI), acetovanillone, acetic anhydride, nitric acid (HNO_3, 65%), ethanol (EtOH, abs.), Boc anhydride and *tert*-butanol were obtained from Sigma Aldrich. Diethyl ether, tetrahydrofuran (THF, abs.), dichloromethane (DCM), and acetonitrile (ACN) were obtained from Fisher Scientific. Potassium carbonate, sodium sulfate, trifluoroacetic acid (TFA) and triethylamine were purchased from Carl Roth. Sodium borohydride, 4-bromobutyric acid ethyl-4-bromobutyrate were obtained from TCI chemicals. Methanol, acetone and sodium hydroxide were obtained from VWR chemicals.

2. Instruments

2.1. Nuclear Magnetic Resonance Spectroscopy (NMR)

NMR measurements were recorded either on an Avance 300 or Avance III 250 spectrometer (Bruker) (300 MHz for 1H-NMR and 75 MHz for ^{13}C-NMR or 250 MHz for ^{1}H-NMR and 63 MHz for ^{13}C-NMR). The chemical shifts are given in parts per million (ppm) relating to the solvent peak of CD_3Cl or DMSO-d6 for ^{1}H-NMRs. The signal splittings are listed as s (singlet), d (doublet), t (triplet), dd (doublet of doublet), m (multiplet), br (broad singlet). The coupling constant (*J*) is stated in Hz. All spectra were analyzed using MestReNova 14.0.0-23239.

2.2. High-Performance Liquid Chromatography (HPLC)

HPLC runs for purification were conducted in preparative scale using a Shimadzu system with the following modules: DGU-20A5R, LC-20AP, CBM-20A, SPD-M20A, SIL-10AP, FRC-18A. The column Gemini NX-C18-110Å, Phenomenex, 150 mm × 30 mm, 5 μm pore size and

a flow rate of 25 mL/min was used. An acetonitrile/water mixture with additional 0.1%TFA was used as eluent with a programmed gradient running from 0% to 100% ACN in water in 45 min (PCL) and 35 min (NCL). The peptides were dissolved in MilliQ water with 0.1% TFA to a concentration of approx. 0.1–0.01 mg/mL and filtered via a PES syringe filter with a pore size of 0.45 μm before injecting approx. 10 mL per run in HPLC and were detected at 214 nm and 254 nm.

Analytical scale HPLC was performed using a Shimadzu system with following modules: DGU-20A5R, LC-20AT, CBM-20A, SPD-M20A, SIL-10ACHT, CTO-20AC. In analytical scale, the column Zorbax XDB-C18, 9.4 × 250 mm, 5 μm pore size was used. The eluent was a gradient from 5% to 80% ACN in water over 22 min (NCL).The peptides were dissolved in MilliQ water with 0.1% TFA to a concentration of 1 mg/mL and filtered via a PES syringe filter with a pore size of 0.45 μm before injecting approx. 50 μL per run in HPLC.

2.3. Liquid Chromatography - Mass Spectrometry (LC-MS)

HPLC-ESI-MS was measured on a Shimadzu LC-2020 Single Quadrupole MS instrument equipped with the modules LC-20AD, SIL-20ACHT, SPD-20A, CTO-using a Kinetex EVO C18 100 Å LC 50 × 2.1 mm column with 2.6 μm pore size. An acetonitrile/water mixture with additional 0.1% formic acid was used as eluent. The gradient was from 5% ACN to 95% ACN over 20 min. The data was processed with LabSolutions and Origin. Samples were dissolved either in methanol or MilliQ water to a concentration of 0.1 mg/mL.

2.4. Matrix-Assisted Laser Desorption/Ionization - Time of Flight Mass Spectrometry (MALDI-ToF MS)

MALDI-ToF measurements were measured on a rapifleX MALDI-TOF/TOF from Bruker and MALDI Synapt G2-SI from Waters. The samples were prepared via dried droplet method by mixing with a saturated α-cyano-4-hydroxycinnamic acid (HCCA) solution in water/ACN 1:1+0.1% TFA before measurement. Appr. 1 μL of the solution was added to the target plate and allowed to dry. The data was evaluated with mMass software.

2.5. Matrix-Assisted Laser Desorption/Ionization - Time of Flight Mass Spectrometry Imaging (MALDI-MSI)

Since the wavelength of the laser (355 nm) is close to the UV-radiation used for fragmentation of the PCL (365 nm), particular care was taken in adjusting the laser intensity. The laser power, which is typically applied for MALDI-MSI of 10^8 W resulted in in-source-fragmentation and a significant contribution to the yield of the RGD-fragment signal was observed. The reduction of the laser intensity to 35%, which is close to desorption/ionization threshold of the HCCA matrix, suppressed in-source fragmentation almost completely.

2.6. Microwave Peptide Synthesizer

Peptides were synthesized using an automated microwave peptide synthesizer (CEM, Liberty Blue™) according to the standard coupling strategy (see chapter 3.3.).

3. Synthesis

3.1. Synthesis of Photo-cleavable Linker, PCL **(8)**, according to Sur et al.[1]

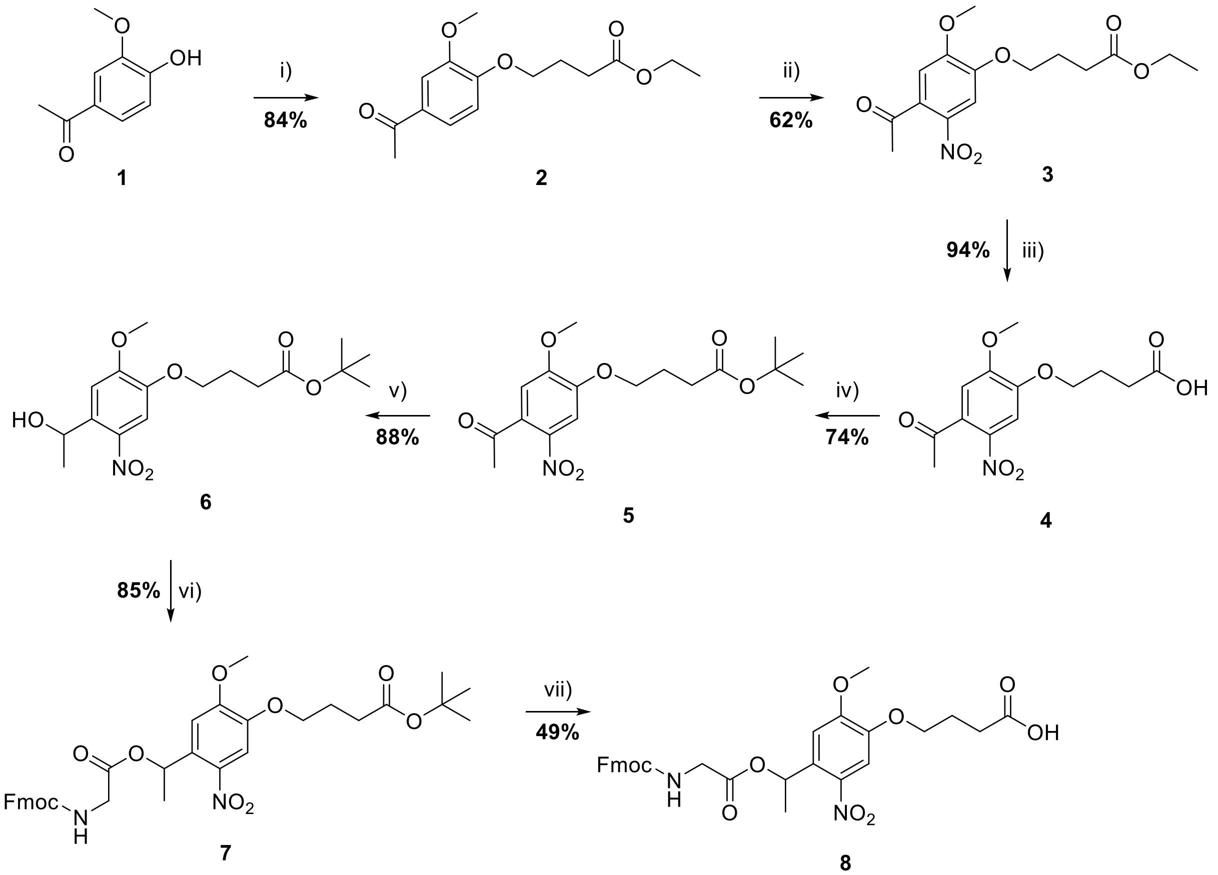

*Scheme S1: Synthesis of **8**: i) Ethyl 4-bromobutanoate, K_2CO_3, DMF, 24 h, rt; ii) HNO_3, Ac_2O, 4 min, 0 °C; iii) EtOH, 1 h, 40 °C; iv) Di-tert-butylpyrocarbonate, DMAP, ^{t}BuOH/THF (3:1, v/v), 21 h, rt; v) $NaBH_4$, EtOH, 5 h, rt; vi) ([(9H-fluoren-9-ylmethoxy)carbonyl]glycine), EDC, DMAP, 1.5 h, rt; vii) TFA, CH_2Cl_2, 4 h, rt.*

(2) Ethyl 4-(4-acetyl-2-methoxyphenoxy)butanoate

To a solution of ethyl 4-bromobutyrate (5.60 mL, 39.23 mmol) in DMF (50 mL) was added 1-(4-hydroxy-3-methoxyphenyl)ethan-1-one (8.03 g, 48.36 mmol) and K_2CO_3 (12.55 g, 90.88 mmol). The reaction mixture was stirred overnight at room temperature and was diluted with water (50 mL) and EtOAc (50 mL). The aqueous phase was extracted three times with EtOAc (50 mL). The combined organic phase was washed three times with water (25 mL) and with brine (25 mL). The organic phase was dried over Na_2SO_4, filtered, and the solvent was removed under reduced pressure to yield a white solid (11.40 g, 40.70 mmol, 84% yield).

Chemical formula C15H20O5, M = 280.32 g/mol

R_F (*n*-hexane/ EtOAc, 5/3) = 0.5

^{1}H-NMR (250 MHz, $CDCl_3$, 25 °C) δ/ppm: 7.57 – 7.50 (m, 2H, H^1, H^3), 6.88 (d, J = 8.2 Hz, 1H, H^4), 4.18 - 4.09 (m, 4H, H^{14}, H^{19}), 3.90 (s, 3H, H^8), 2.55 (s, 3H, H^{11}), 2.53 (s, 2H, H^{15}), 2.18 (q, J = 6.8 Hz, 2H, H^{13}), 1.25 (t, J = 7.1 Hz, 3H, H^{20}).

^{13}C-NMR (63 MHz, $CDCl_3$, 25 °C) δ/ppm: 196.97, 173.15, 152.72, 149.35, 130.58, 123.34, 111.30, 110.49, 67.89, 60.63, 56.10, 30.68, 26.36, 24.38, 14.34.

LC-MS: (ESI+) *m/z* calc. for $C_{15}H_{20}O_5$ $[M+H]^+$, 281.32 g/mol; found 281.15 g/mol.

(3) Ethyl 4-(4-acetyl-2-methoxy-5-nitrophenoxy)butanoate

To a solution of ethyl 4-(4-acetyl-2-methoxyphenoxy)butanoate **(2** (10.35 g, 36.92 mmol) in 175 mL acetic anhydride at 0 °C was added 65% HNO_3 (6 mL) dropwise over 2 min. The brownish-yellow solution was added to ice-water (1500 mL) 4 min. after starting to add HNO_3. The reaction mixture was stirred for further 30 min. The yellow solid was filtered and dissolved in dichloromethane. The organic phase was washed three times with sat. aq. $NaHCO_3$ (150 mL) and water (150 mL). The organic phase was dried over Na_2SO_4, filtered, and the solvent was

removed under reduced pressure. The crude product showed 30% of side product due to nitration side reactions and was purified via column chromatography eluting with 1.5% MeOH in CH_2Cl_2 to yield a yellow solid (7.52 g, 23.1 mmol, 63% yield).

Chemical formula C15H19N5O7. M = 325.11 g/mol.

R_F (CH_2Cl_2 with 1.5% MeOH) = 0.20

^{1}H-NMR (250 MHz, $CDCl_3$, 25 °C) δ/ppm: 7.61 (s, 1H, H^1), 6.74 (s, 1H, H^4), 4.22 – 4.10 (m, 4H, H^{14}, H^{19}), 3.96 (s, 3H, H^8), 2.55 (t, J = 7.2 Hz, 2H, H^{13}), 2.49 (s, 3H, H10), 2.20 (q, J = 6.7 Hz, 2H, H15), 1.27 (t, J = 7.1 Hz, 3H, H20).

^{13}C-NMR (63 MHz, $CDCl_3$, 25 °C) δ/ppm: 200.17, 172.82, 154.30, 148.85, 132.87, 108.72, 107.95, 68.46, 60.63, 56.61, 30.51, 30.44, 24.17, 14.24.

LC-MS: (ESI+) m/z calc. for $C_{15}H_{19}N_5O_7$ $[M+H]^+$, 326.12 g/mol; found 326.15 g/mol.

(4) 4-(4-Acetyl-2-methoxy-5-nitrophenoxy)butanoic acid

To a suspension of ethyl-4-(4-acetyl-2-methoxy-5-nitrophenoxy)butanoate **(3)** (3.65 g, 11.22 mmol) in EtOH (50 mL) was given 1M aq. NaOH (20 mL). The reaction mixture was stirred at 40 °C for 1.5h. The solvent was removed under reduced pressure, and the aqueous residue was taken up in sat. aq. $NaHCO_3$. The aqueous layer was washed three times with CH_2Cl_2 (50 mL) and acidified with conc. HCl to pH 1. A brown-yellow precipitation was observed. The aqueous layer was extracted three times with CH_2Cl_2 (150 mL), and the organic layer was washed two times with 0.5 N HCl (100 mL). The organic phase was dried over Na_2SO_4, filtered and the solvent was removed under reduced pressure to obtain a yellow solid (3.19 g, 10.75 mmol, 94% yield).

Chemical formula C13H15NO7. M = 297.08 g mol^{-1}.

R_F (CH_2Cl_2 with 10% MeOH) = 0.62.

^1H-NMR (250 MHz, $CDCl_3$, 25 °C) δ/ppm: 7.62 (s, 1H, H^1), 6.75 (s, 1H, H^4), 4.18 (t, J = 6.1 Hz, 2H, H^{14}), 3.95 (s, 3H, H^8), 2.63 (t, J = 7.1 Hz, 2H, H^{13}), 2.50 (s, 3H, H^{10}), 2.22 (p, J = 6.7 Hz, 2H, H^{15}).

^{13}C-NMR (63 MHz, $CDCl_3$, 25 °C) δ/ppm: 201.05, 175.23, 154.28, 148.85, 138.09, 132.51, 108.53, 107.90, 77.67, 77.16, 76.65, 68.34, 56.41, 30.16, 30.01, 23.97.

LC-MS: (ESI+) *m/z* calc. for $C_{13}H_{15}NO_7$ $[M+Na]^+$, 320.07 g/mol; found 320.10 g/mol.

(5) *tert*-Butyl 4-(4-acetyl-2-methoxy-5-nitrophenoxy)butanoate

To a suspension of 4-(4-acetyl-2-methoxy-5-nitrophenoxy)butanoic acid **(4)** (3.00 g, 10.43 mmol) in 80 mL ^{t}BuOH/THF solution (3:1 v/v) was added DMAP (382 mg, 3.13 mmol). Di-*tert*-butylpyrocarbonate (6.83 g, 31.29 mmol) was added portion wise over 1.5 h. Bubbling was observed, and the flask was equipped with a balloon under argon atmosphere. After stirring for 21 h, TLC (10% MeOH in CH_2Cl_2) showed complete conversion. The solvent was removed under reduced pressure to obtain an orange oil. The residue was taken up in CH_2Cl_2 (100 mL), washed twice with 0.2 N HCl (50 mL) and three times with sat. aq. $NaHCO_3$ (50 mL). The organic layer was then dried over Na_2SO_4. The solvent was removed under reduced pressure to afford an orange oil, which was further purified through column chromatography eluting with 2% MeOH in CH_2Cl_2 to afford a yellow viscous oil (2.76g, 7.81 mmol, 74% yield).

Chemical formula $C_{17}H_{23}NO_7$. M = 353.17 g/mol.

R_F (CH_2Cl_2 with 2% MeOH) = 0.56.

^1H-NMR (250 MHz, $CDCl_3$, 25 °C) δ/ppm: 7.61 (s, 1H, H^1), 6.74 (s, 1H, H^4), 4.14 (t, J = 6.4 Hz, 2H, H^{14}), 3.96 (s, 3H, H^{11}), 2.47 (d, J = 9.6 Hz, 5H, H^{15}, H^8), 2.15 (p, J = 6.9 Hz, 2H, H^{16}), 1.45 (s, 9H, H^{21}–H^{23}).

^{13}C-NMR (63 MHz, $CDCl_3$, 25 °C) δ/ppm: 200.18, 172.12, 154.30, 148.92, 138.37, 132.83,

108.73, 107.99, 80.68, 77.54, 77.04, 76.53, 68.63, 56.62, 31.69, 30.44, 28.12, 24.31.

LC-MS: (ESI-) *m/z* calc. for $C_{17}H_{23}NO_7$ $[M]^-$, 353.14 g/mol; found 353.15 g/mol.

(6) *tert*-Butyl 4-(4-(1-hydroxyethyl)-2-methoxy-5-nitrophenoxy)butanoate

$NaBH_4$ (1.03 g, 27.2 mmol) was added to a suspension of *tert*-butyl-4-(4-acetyl-2-methoxy-5-nitrophenoxy)butanoate **(5)** (2.76 g, 7.81 mmol) in EtOH (45 mL). After 5 h the reaction was quenched by adding a citric acid solution (600 mg/mL) until pH 4 is reached. The solution was then diluted with CH_2Cl_2 (100 mL) and water (50 mL). The organic layer was washed with brine and dried over Na_2SO_4. The solvent was removed under reduced pressure to yield a viscous orange oil (2.46 g, 6.98 mmol, 88%).

Chemical formula $C_{17}H_{25}NO_7$. M = 355.16 g/mol.

R_F (CH_2Cl_2 with 2% MeOH) = 0.32.

^{1}H-NMR (250 MHz, $CDCl_3$, 25 °C) δ/ppm: 7.55 (s, 1H, H^1), 7.28 (s, 1H, H^4), 5.54 (q, J = 6.3 Hz, 1H, H^9), 4.08 (t, J = 6.4 Hz, 2H, H^{15}), 3.96 (s, 3H, H^8), 2.46 (dt, J = 14.5, 7.3 Hz, 2H, H^{14}), 2.13 (q, J = 6.6 Hz, 2H, H^{16}), 1.53 (d, J = 6.2 Hz, 3H, H^{10}), 1.44 (s, 9H, H^{21}–H^{23})..

^{13}C-NMR (63 MHz, $CDCl_3$, 25 °C) δ/ppm: 172.27, 154.12, 146.96, 139.51, 136.94, 109.05, 108.65, 80.60, 77.56, 77.05, 76.54, 68.42, 65.75, 56.36, 31.82, 28.11, 24.42, 24.29, 21.85.

LC-MS: (ESI+) *m/z* calc. for $C_{17}H_{25}NO_7$ $[M+Na]^+$, 378.15 g/mol; found 378.15 g/mol.

(7) *tert*-Butyl 4-(4-(1-((([(9*H*-fluoren-9-ylmethoxy)carbonyl]glycyl)oxy)ethyl)-2-methoxy-5-nitrophenoxy)butanoate

To [(9*H*-fluoren-9-ylmethoxy)carbonyl]glycine (3.18 g, 10.5 mmol), EDC × HCl (2.38 g, 12.3 mmol) and DMAP (90 mg, 0.7 mmol) was added dry CH_2Cl_2 (20 mL). The suspension becomes clear solution after stirring for 5 min and *tert*-butyl 4-(4-(1-hydroxyethyl)-2-methoxy-5-

nitrophenoxy)butanoate **(6)** (2.40 g, 6.75 mmol) was added in CH_2Cl_2 (13 mL). After stirring for 4 h at room temperature, the reaction mixture was washed twice with water (50 mL) and twice with sat. aq. $NaHCO_3$ (100 mL). The organic layer was dried over Na_2SO_4. The solvent was removed under reduced pressure to afford a yellow powder that was further purified via column chromatography, eluting with 2% MeOH in CH_2Cl_2, yielding a light yellow solid with small amount of impurities (3.65 g, 5.76 mmol, 85%).

Chemical formula $C_{34}H_{38}N_2O_{10}$. M = 634.24 g/mol.

R_F (CH_2Cl_2 with 2% MeOH) = 0.54, R_F (Cyclohexane/ EtOAc, 5/3) = 0.52.

^{1}H-NMR (250 MHz, $CDCl_3$, 25 °C) δ/ppm: 7.75 (d, J = 7.5 Hz, 2H, H^{36}, H^{37}), 7.57 (d, J = 6.7 Hz, 3H, H^{33}, H^{40}), 7.44 – 7.20 (m, 4H, H^{34}, H^{35}, H^{38}, H^{39}), 6.98 (s, 1H, H^1), 6.55 (q, J = 6.2 Hz, 1H, H^4), 5.30 (d, J = 5.2 Hz, 1H, H^9), 4.47 – 4.27 (m, 2H, H^{41}), 4.23 – 3.95 (m, 5H, H^{31}, H^{14}, H^{19}), 3.94 (s, 3H, H^{10}), 2.43 (t, J = 7.3 Hz, 2H, H^{18}), 2.13 (h, J = 7.1, 5.7 Hz, 2H, H^{20}), 1.64 (d, J = 6.4 Hz, 3H, H^8), 1.45 (s, 9H, H^{25}–H^{27}).

^{13}C-NMR (63 MHz, $CDCl_3$, 25 °C) δ/ppm: 172.21, 169.03, 156.30, 154.18, 147.44, 143.72, 143.69, 141.31, 141.27, 139.64, 132.41, 127.77, 127.09, 125.04, 120.03, 108.94, 108.01, 80.61, 69.79, 68.41, 67.27, 56.42, 47.03, 42.95, 31.79, 28.13, 24.39, 21.98.

LC-MS: (ESI+) *m/z* calc. for $C_{34}H_{38}N_2O_{10}$ $[M+Na]^+$, 657.24 g/mol; found 657.25 g/mol.

(8) 4-(4-(1-((([(9*H*-fluoren-9-ylmethoxy)carbony]glycyl)oxy)ethyl)-2-methoxy-5-nitrophenoxy)butanoic acid

To a solution of *tert*-butyl 4-(4-(1-(2-(Fmoc-amino)acetoxy)ethyl)-2-methoxy-5-nitrophenoxy)butanoate **(7)** (500 mg, 0.787 mmol) in CH_2Cl_2 (5 mL) was added TFA (0.75 mL). The light yellow suspension becomes a deep yellow clear solution. After stirring for 5 h at room temperature, the solvent was evaporated under reduced pressure. The residue was taken up with CH_2Cl_2 and was washed twice with 0.5 N HCl (40 mL). The organic layer was dried

over Na_2SO_4. The solvent was removed under reduced pressure to afford a yellow powder that was further purified via column chromatography, eluting with 4% MeOH in CH_2Cl_2, yielding a light yellow solid (224 mg, 0.387 mmol, 49%).

Chemical formula $C_{30}H_{30}N_2O_{10}$. *M* = 578.57 g/mol.

R_F (CH_2Cl_2 with 4% MeOH) = 0.33.

^{1}H-NMR (250 MHz, $CDCl_3$, 25 °C) *δ*/ppm: 7.75 (d, *J* = 7.5 Hz, 2H, H^{33}, H^{34}), 7.55 (d, *J* = 6.7 Hz, 2H, H^{30}, H^{37}), 7.42 – 7.26 (m, 4H, H^{31}, H^{32}, H^{35}, H^{36}), 6.98 (s, 1H, H^1), 6.53 (q, *J* = 6.4 Hz, 1H, H^4), 5.30 (t, *J* = 5.8 Hz, 1H, H^9), 4.39 – 4.23 (m, 2H, H^{38}), 4.17 – 4.00 (m, 5H, H^{28}, H^{20}, H^{14}), 3.94 (s, 3H, H^8), 2.60 (t, *J* = 7.3 Hz, 2H, H^{19}), 2.17 (h, *J* = 7.1, 5.7 Hz, 2H, H^{21}), 1.64 (d, *J* = 6.3 Hz, 2H, H^{10}).

^{13}C-NMR (63 MHz, $CDCl_3$, 25 °C) *δ*/ppm: 178.02, 169.08, 156.36, 154.20, 147.30, 143.68, 141.27, 139.63, 132.55, 127.77, 127.09, 125.03, 120.01, 109.03, 108.09, 69.79, 68.03, 67.30, 56.39, 47.03, 42.96, 30.16, 23.94, 21.96.

LC-MS: (ESI+) *m/z* calc. for $C_{30}H_{30}N_2O_{10}$ $[M+Na]^+$, 601.17 g/mol; found 601.20 g/mol.

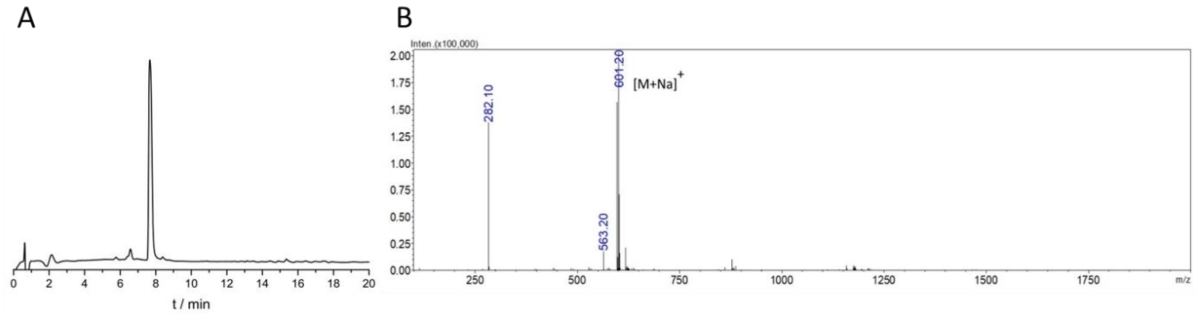

*Figure S1: A) LC-MS chromatogram (detector: 214 nm) of compound **(8)**. B) MS (ESI+) m/z, calc. for $C_{30}H_{30}N_2O_{10}$ $[M+Na]^+$, 601.17 g/mol; found 601.20 m/z.*

3.2. Synthesis of non- photo cleavable linker NCL **(14)**, according to Sur et al.[1]

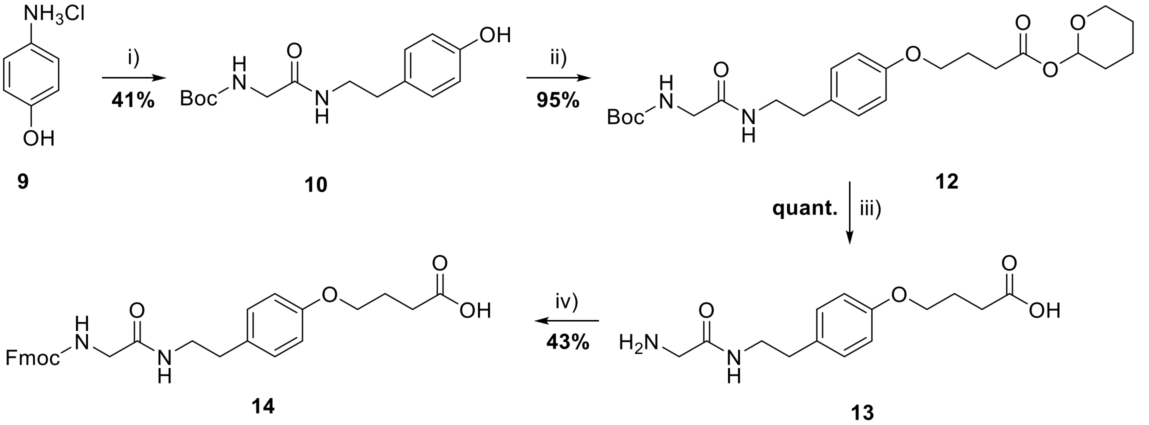

*Scheme S2: Synthesis of **14**. i) (tert-butoxycarbonyl)glycine, EDC, DMAP, NEt_3, THF, 6 h, reflux; ii) tetrahydro-2H-pyran-2-yl 4-bromobutanoate **11**, K_2CO_3, KI, acetone, 3 d, reflux; iii) TFA, CH_2Cl_2, 2 h, rt; iv) 1-([(9H-Fluoren-9-ylmethoxy)carbonyl]oxy)-2,5-pyrrolidinedione, NEt_3, H_2O/ACN (1:1 v/v), 4 h, rt. Synthesis of **11** according to literature.[2]*

(10) *tert*-Butyl (2-((4-hydroxyphenethyl)amino)-2-oxoethyl)carbamate

4-Hydroxyphenethylamine hydrochloride (2.00 g, 11.5 mmol) was added to dry THF (20 mL) and NEt_3 (5 mL). To this suspension (*tert*-butoxycarbonyl)glycine (2.26 g, 12.9 mmol) was added, followed by EDC (2.47 g, 12.7 mmol) and DMAP (140 mg, 1.15 mmol). The white suspension was heated at reflux for 6 h, and then the solvent was removed under reduced pressure. The yellow residue was dissolved in CH_2Cl_2 (40 mL), washed three times with water (40 mL) and dried over Na_2SO_4. The solvent was removed to yield a viscous yellow oil that was purified via column chromatography, eluting with 20% MeOH in CH_2Cl_2. The combined organic fractions were evaporated to yield a colorless solid with small impurities. The solid was recrystallized from chloroform and small amount of MeOH. The white crystals were washed with *n*-hexane. A colorless solid was obtained (1.39 g, 4.74 mmol, 41%).

Chemical formula $C_{16}H_{23}NO_4$. M = 293.16 g/mol.

R_F (CH_2Cl_2 with 20% MeOH) = 0.72.

^{1}H NMR (250 MHz, Chloroform-d with 10% CD_3OD) δ 6.95 (d, J = 8.1 Hz, 2H, H^1, H^3), 6.68 (d, J = 8.2 Hz, 2H, H^4, H^6), 3.60 (s, 2H, H^{12}), 3.38 – 3.24 (m, 2H, H^9), 2.64 (t, J = 7.2 Hz, 2H,

H^8), 1.36 (s, 9H, H^{17}–H^{19}).

^{13}C NMR (63 MHz, $CDCl_3$ with 10% CD_3OD) δ 170.11, 156.47, 155.34, 129.67, 115.40, 77.67, 77.16, 76.65, 43.76, 40.97, 34.55, 28.19.

LC-MS: (ESI+) *m/z* calc. for $C_{16}H_{23}NO_4$ $[M+Na]^+$, 317.14 g/mol; found 317.15 g/mol.

(11) Tetrahydro-2*H*-pyran-2-yl 4-bromobutanoate

4-Bromobutanoic acid (2.50 g, 15.00 mmol) and 3,4-dihydro-2H-pyran (1.5 mL, 15.12 mmol) were dissolved in CH_2Cl_2 and cooled down to 0 °C. To this solution was added pyridinium *p*-toluenesulfonate (0.376 g, 1.50 mmol) and stirred at 0 °C for 30 min and at ambient temperature for 4 h. The solution was washed three times with 4% aq. Na_2CO_3 (100 mL) and three times with water (100 mL). The organic layer was then dried over Na_2SO_4. The solvent was removed to yield a viscous colorless oil (3.42 g, 13.68 mmol, 91%).

Chemical formula $C_9H_{15}BrO_3$. M = 251.12 g/mol.

^{1}H-NMR (250 MHz, $CDCl_3$, 25 °C) δ/ppm: 5.99 (d, J = 3.3 Hz, 1H, H^8), 3.95 – 3.62 (m, 2H, H^{10}), 3.48 (t, J = 6.4 Hz, 2H, H^2), 2.56 (t, J = 7.2 Hz, 2H, H^4), 2.19 (p, J = 6.8 Hz, 2H, H^3), 1.88 – 1.51 (m, 6H, H^{11} - H^{13}).

^{13}C-NMR (63 MHz, $CDCl_3$, 25 °C) δ/ppm: 171.49, 92.86, 63.43, 32.87, 32.71, 29.26, 27.70, 25.01, 18.72.

(12) Tetrahydro-2*H*-pyran-2-yl 4-(4-(2-(2-((*tert*-butoxycarbonyl)amino)acetamido)ethyl)phenoxy)butanoate

tert-Butyl (2-((4-hydroxyphenethyl)amino)-2-oxoethyl)carbamate **(10)** (1.39 g, 4.72 mmol) and tetrahydro-2*H*-pyran-2-yl 4-bromobutanoate **(11)** (1.90, 7.08 mmol) were dissolved in acetone (15 mL) in a two necked flask with air condenser and a stirrer. K_2CO_3 and KI were

added to the solution and stirred at 80 °C for three days. The solution was dissolved in EtOAc (50 mL), washed three times with water (50 mL), once with brine (50 mL) and dried over Na_2SO_4. The solvent was removed to yield a viscous yellow oil that was purified via column chromatography, eluting with 4% MeOH in CH_2Cl_2 to obtain a yellow oil (2.09 g, 4.51 mmol, 95%).

Chemical formula $C_{24}H_{36}N_2O_7$. M = 464.56 g/mol.

R_F (CH_2Cl_2 with 4% MeOH) = 0.65.

^{1}H NMR (250 MHz, Chloroform-d) δ 7.08 (d, J = 8.2 Hz, 2H, H^{13}, H^{15}), 6.81 (d, J = 8.3 Hz, 2H, H^{16}, H^{18}), 6.14 (s, 1H, H^{26}), 5.99 (d, J = 3.9 Hz, 1H, H^7), 5.13 (s, 1 H, H^{22}), 4.04 - 3.80 (m, 2H, H^9), 3.73 (d, J = 5.9 Hz, 2H, H^{20}), 3.47 (q, J = 6.7 Hz, 2H, H^{21}), 2.73 (t, J = 7.0 Hz, 2H, H^1), 2.56 (t, J = 7.3 Hz, 2H, H^3), 2.12 (p, J = 6.7 Hz, 2H, H^2), 1.84 - 1.51 (m, 6H, H^{10} - H^{12}), 1.43 (s, 9H, H^{29} - H^{31}).

^{13}C NMR (63 MHz, $CDCl_3$) δ 172.00, 169.30, 157.53, 130.70, 129.67, 114.64, 92.63, 66.64, 63.31, 40.76, 34.73, 30.99, 29.19, 28.30, 24.92, 24.53, 18.66.

LC-MS: (ESI+) *m/z* calc. for $C_{24}H_{36}N_2O_7$ $[M+Na]^+$, 487.24 g/mol; found 487.25 g/mol.

(13) 4-(4-(2-(2-Aminoacetamido)ethyl)phenoxy)butanoic acid

Tetrahydro-2*H*-pyran-2-yl 4-(4-(2-(2-((*tert*-butoxycarbonyl)amino)acetamido)ethyl)phenoxy)-butanoate **(12)** (2.09 g, 4.50 mmol) was dissolved in a mixture of TFA (7.87 mL) and CH_2Cl_2 (31.00 mL) and stirred at ambient temperature for 120 min. The solvent was removed under reduced pressure. The residue was dissolved in Et_2O (100 mL) by sonicating for 10 min. A white precipitate was observed. The product was recovered by filtration. The solids were taken up in CH_2Cl_2 with 20% MeOH and dried over Na_2SO_4. After removing the solvent under reduced pressure, a white powder was obtained (1.25 g, 4.45 mmol, quant.).

Chemical formula $C_{14}H_{20}N_2O_4$. M = 280.32 g/mol.

^{1}H NMR (250 MHz, DMSO-d6) δ 6.95 (d, J = 8.2 Hz, 2H, H^7, H^9), 6.68 (d, J = 8.2 Hz, 2H, H^{10}, H^{12}), 3.84 (t, J = 6.1 Hz, 2H, H^{15}), 3.42 (s, 2H, H^{18}), 3.36 – 3.18 (m, 2H, H^{14}), 2.60 (t, J = 7.4 Hz, 2H, H^1), 2.35 (t, J = 7.2 Hz, 2H, H^3), 1.93 (p, J = 6.7 Hz, 2H, H^2).

^{13}C NMR (63 MHz, DMSO-d6) δ 175.83, 165.69, 157.40, 130.57, 129.47, 114.44, 66.68, 49.73, 41.07, 40.18, 34.30, 30.41, 24.46.

(14) 4-(4-(2-(2-([(9*H*-fluoren-9-ylmethoxy)carbonyl]amino)acetamido)ethyl)phenoxy)butanoic acid

4-(4-(2-(2-Aminoacetamido)ethyl)phenoxy)butanoic acid **(13)** (1.53 g, 5.46 mmol) was dissolved in a mixture of H_2O (12.4 mL) and NEt_3 (1.86 mL). 1-([(9*H*-Fluoren-9-ylmethoxy)carbonyl]oxy)-2,5-pyrrolidinedione (2.26 g, 6.71 mmol) was dissolved by gentle heating in acetonitrile (12.4 mL) and added to the aqueous solution. After stirring the reaction mixture at ambient temperature for 4 h the reaction mixture was concentrated to 10 mL. To the remaining solution 5% aq. citric acid (60 mL) was added. The mixture was extracted 4 times with 10% MeOH in CH_2Cl_2 (50 mL) and the combined organic layers were dried over Na_2SO_4 and the solvent was removed under reduced pressure. The product was purified by recrystallization from EtOH (200 mL) to yield a white solid (1.08 g, 2.16 mmol, 40%).

Chemical formula $C_{29}H_{30}N_2O_6$. M = 502.557 g/mol.

^{1}H NMR (250 MHz, DMSO-d6) δ 12.10 (s, 1H, H^{18}) 7.87 (t, J = 8.2 Hz, 2H, H^{29}, H^{30}), 7.71 (d, J = 7.4 Hz, 2H, H^{26}, H^{33}), 7.54-7.26 (m, 4H, H^{27}, H^{28}, H^{31}, H^{32}), 7.09 (d, J = 8.1 Hz, 2H, H^1, H^3), 6.82 (d, J = 8.1 Hz, 2H, H^4, H^6), 4.29 (d, J = 6.8 Hz, 2H, H^{34}), 4.20 (d, J = 7.5, 1H, H^{24}), 3.91 (t, J = 6.5 Hz, 2H, H^9) 3.56 (d, J = 6.0 Hz, 2H, H^{11}), 3.23 (t, J = 6.9 Hz, 2H, H^8), 2.63 (t, J = 7.4 Hz, 2H, H^{14}), 2.36 (t, J = 7.4 Hz, 2H, H^{16}), 1.90 (p, J = 6.9 Hz, 2H, H^{15}).

^{13}C NMR (63 MHz, DMSO-d6) δ 174.54, 169.31, 157.38, 156.90, 144.31, 141.18, 131.74, 130.01, 128.09, 127.52, 125.71, 120.56, 114.78, 66.93, 66.17, 47.12, 43.97, 40.92, 34.76, 30.59, 24.75.

LC-MS: (ESI+) *m/z* calc. for $C_{29}H_{30}N_2O_6$ $[M+Na]^+$, 525.20 g/mol; found 525.25 g/mol.

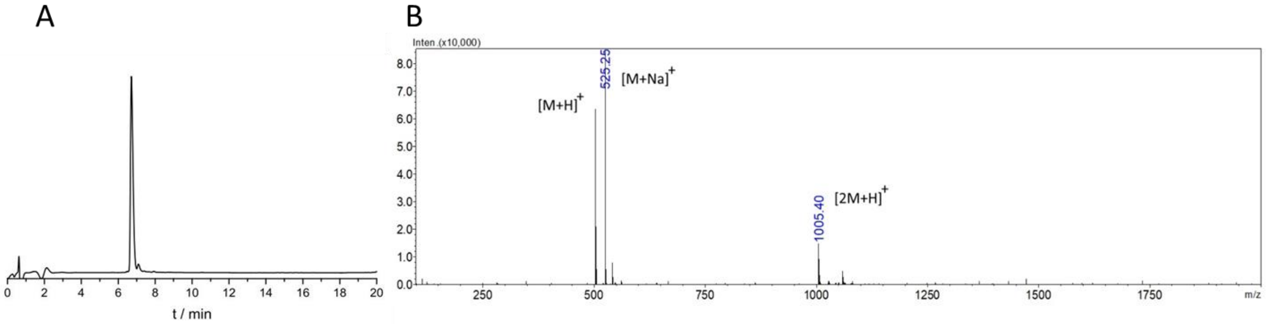

*Figure S2: A) LC-MS chromatogram (detector: 214 nm) of compound **(14)**. B) MS (ESI+) m/z calc. for $C_{29}H_{30}N_2O_6$ $[M+Na]^+$, 525.20 g/mol; found 525.25 m/z.*

3.3. Standard Peptide Synthesis Strategy

SPPS-grade solvents were utilized together with Fmoc-L-Phe-Wang resin for Fmoc amino acid loading (0.65 mmol/g). The beads were swollen in DMF while shaking the reaction vessel for 1h. After draining the DMF a piperidine solution (20% in DMF) was added to the vessel, which was microwaved at 155 W, 75 °C for 15 s and at 30 W, 90 °C for 50 s. Afterwards the piperidine solution was sucked off and the beads were washed three times with DMF. After the addition of the Fmoc-protected amino-acid (5 eq relative to the resin loading capacity) in DMF, DIC (5 eq) in DMF and Oxyma Pure® (10 eq) in DMF were added to the reaction vessel. After microwaving at 170 W, 75 °C for 15 s and at 30 W, 90 °C for 110 s the solution was removed and the beads were washed with DMF. This procedure was repeated for each amino acid. In the final step, the Fmoc-cleavage was performed by microwaving at 155 W, 75 °C for 15 s and at 30 W, 90 °C for 50 s with piperidine solution (20% in DMF). The resin was washed manually with DCM after Fmoc-cleavage. The peptide was cleaved off the resin through treatment with

2 mL of trifluoroacetic acid (TFA) containing 2.5% water and 2.5% triisopropylsilane (TIPS) for 2 h. This solution was added to cold diethyl ether (40 mL) and afterwards centrifuged at 3000 rpm for 15 min to afford a white precipitate.

3.4. Irradiation

Irradiation of samples were conducted with LED from Opulent Americas (Starboard Luminus SST-10-UV-A130) with a peak wave length at 365 nm and a current of 1 A. The emission spectrum of the LED was measured via an Ulbricht sphere as shown in Fig. 3.

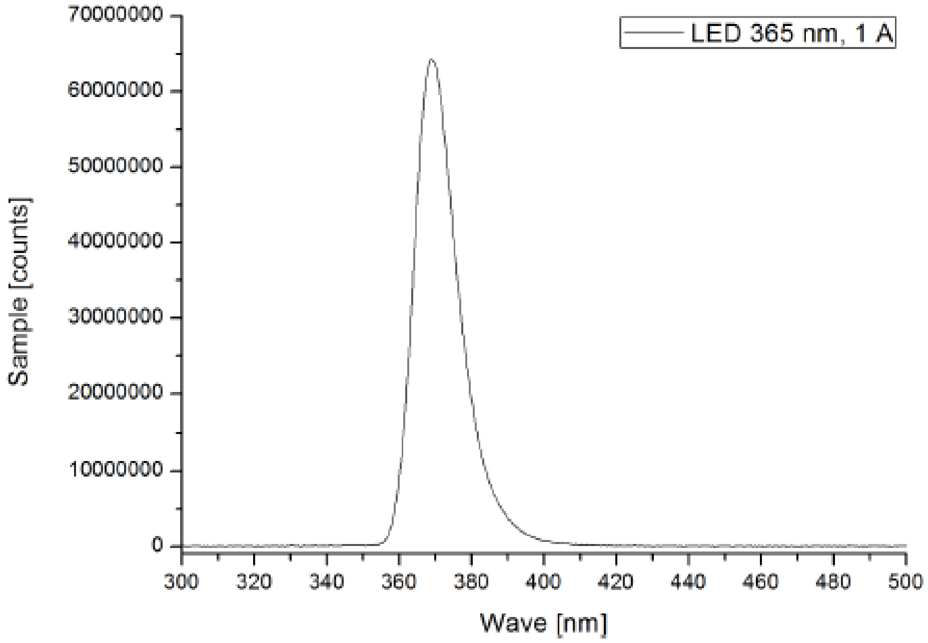

Figure S3: Emission spectrum of LED (Opulent Americas) at 365 nm, 1 A and a radiant flux of 875 mW.

4. Additional Characterization RGD-NCL-CKFKFQF **(16)**

4.1. LC-MS

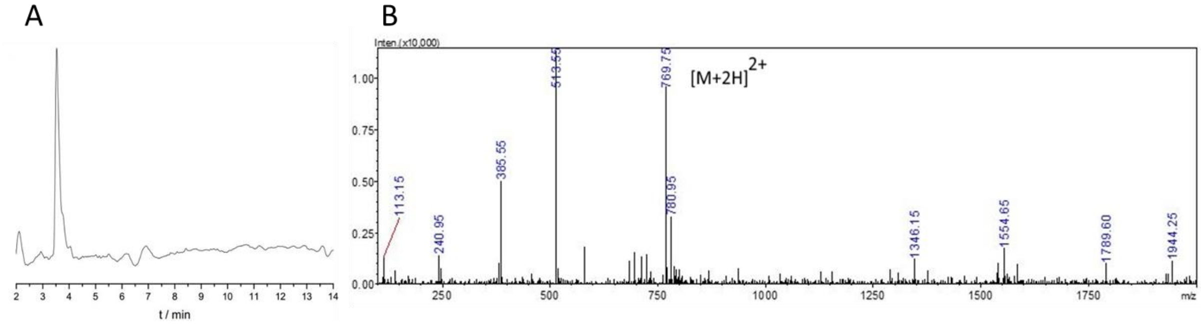

*Figure S4: A) LC-MS chromatogram (detector: 214 nm) of RGD-NCL-CKFKFQF **(16)**. B) MS (ESI+) m/z calc. for C73H104N18O18S [M+2H]2+, 769.65 g mol-1; found 769.75 m/z*

4.2. TEM

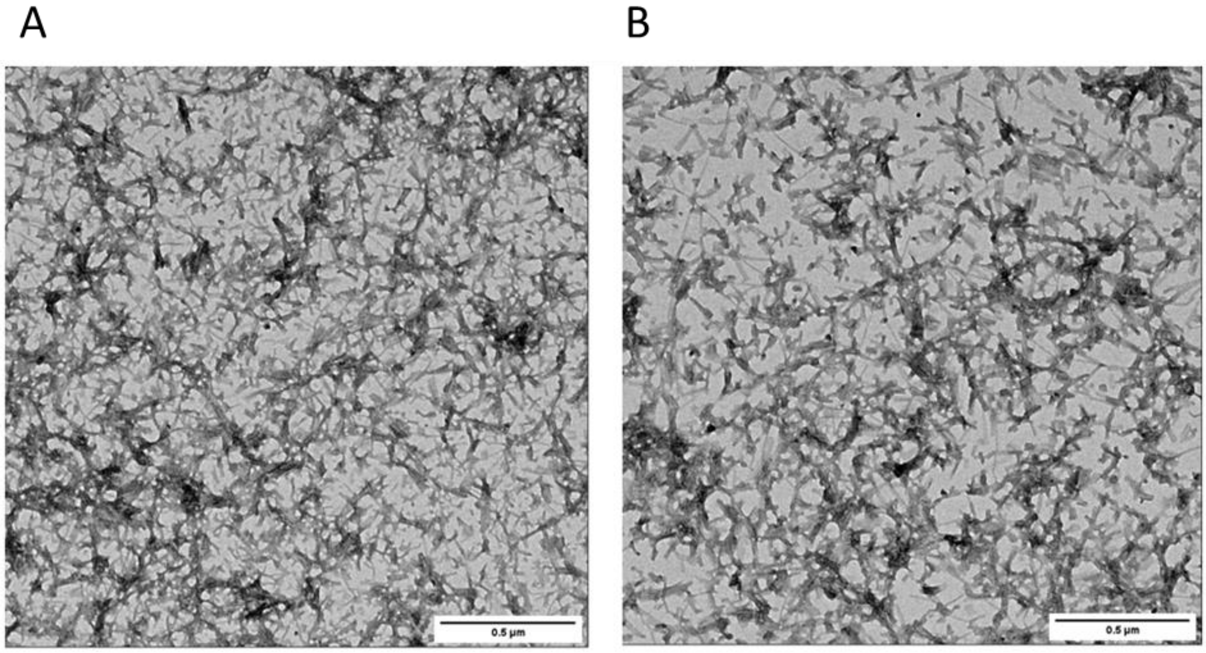

*Figure S5: TEM images of RGD-NCL-CKFKFQF amyloid nanofibers formed in solution. A) Aqueous peptide solution without UV irradiation and incubation for 24h to form fibrils **(16)** and B) aqueous peptide solution after UV irradiation and subsequent incubation for 24h to form fibrils **(16-UV)** (scalebars: 500 nm).*

4.3. ThT-Assay

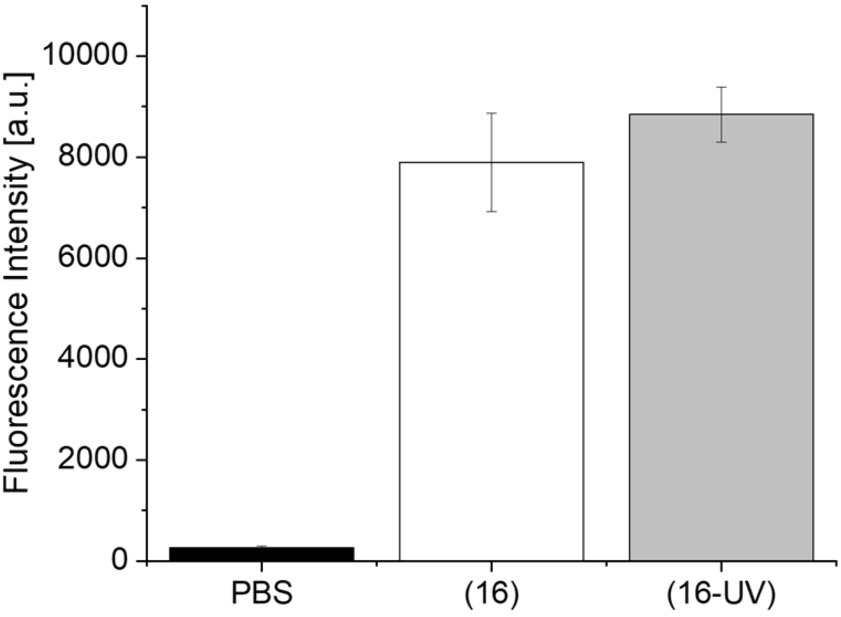

Figure S6: ThT assay shows high fluorescence for nanofibers before ***(16)*** *and after UV irradiation* ***(16-UV)****. As control PBS was used.*

4.4. Fourier-Transform Infrared Spectroscopy (FT-IR)

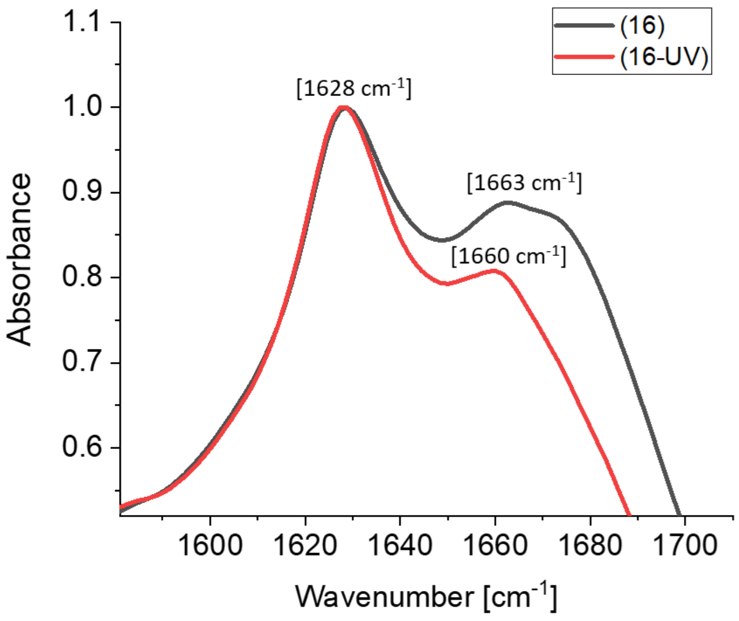

*Figure S7: FT-IR spectra of peptide **(16)** structures show characteristic amyloid signals for both UV treated peptide (**16-UV**: red line) and non-treated (**16**: black line) samples.*

4.5. HPLC study

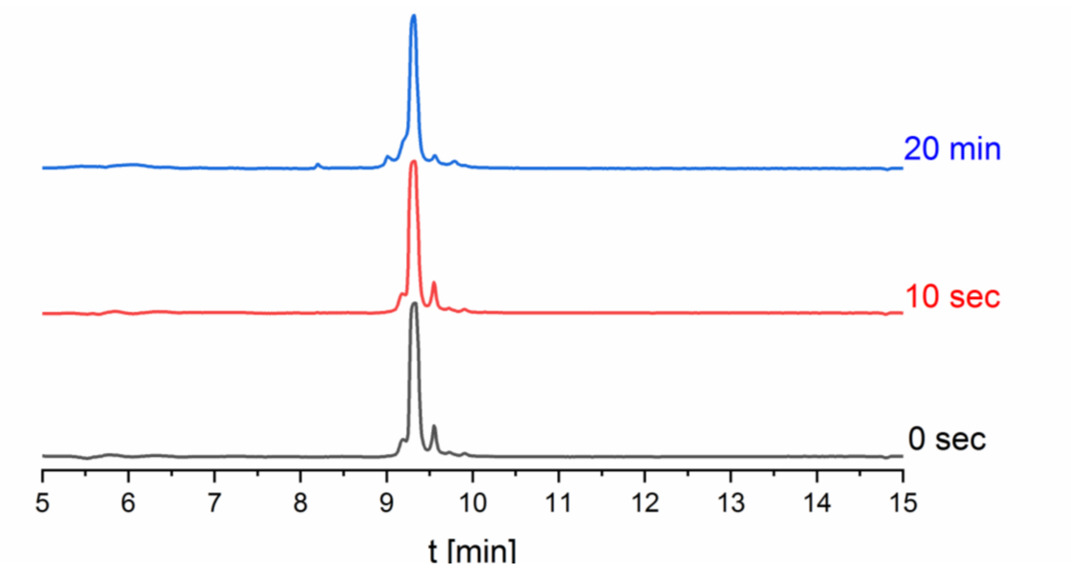

*Figure S8: HPLC measurement (detector 190 nm) of RGD-NCL-CKFKFQF **(16)** without irradiation (0 sec) and after UV irradiation for 10 sec and 20 min.*

4.6. SEM

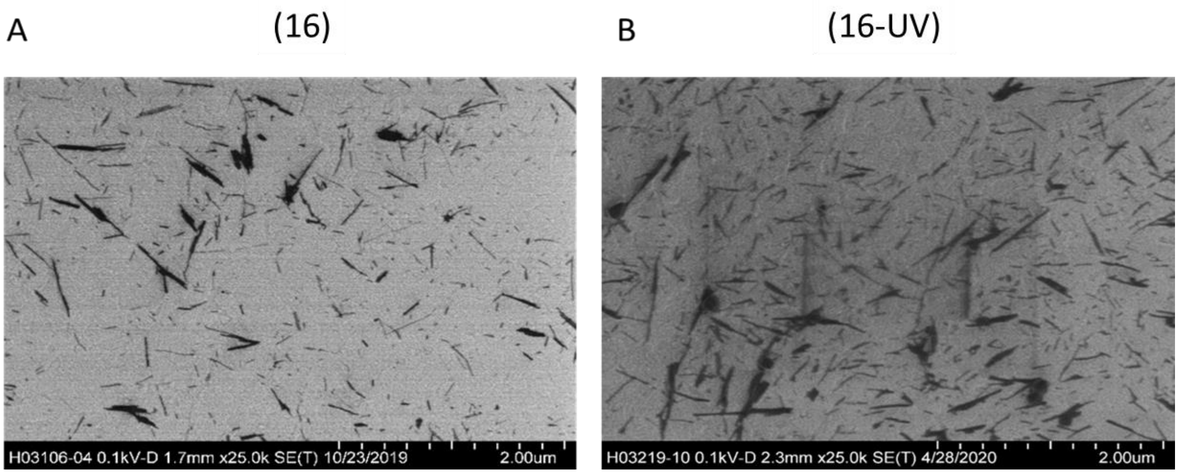

*Figure S9: SEM images of RGD-NCL-CKFKFQF **(16)** amyloid nanofibers formed in solution. A) Sample **(16)** before irradiation and B) sample **(16-UV)** after irradiation (scalebars: 2 μm).*

4.7. ProteoStat-Assay on Surface

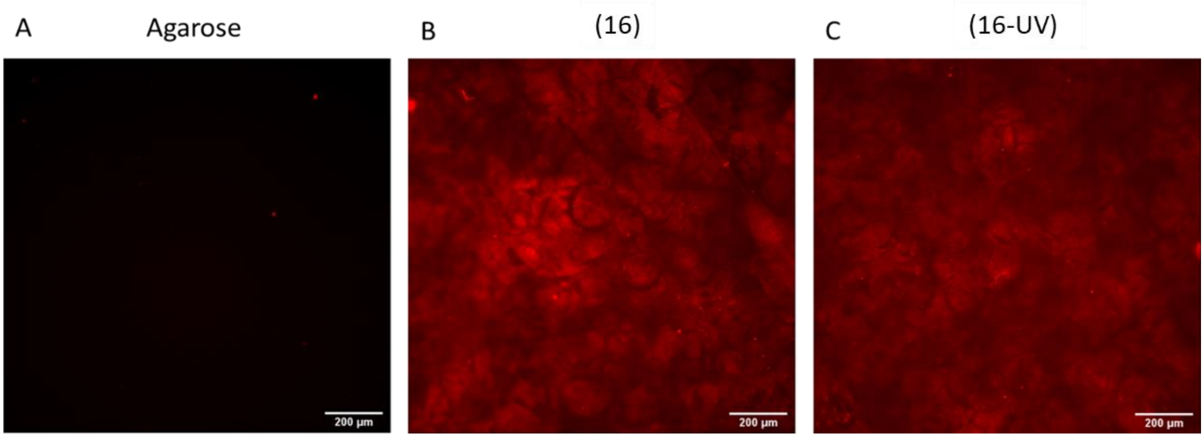

*Figure S10: The Proteostat staining shows a homogeneous coating over a larger area for both, irradiated (**16-UV**, C) and non-irradiated samples (**16**, B). A) Control sample with only agarose coating (scalebars: 200 μm).*

4.8. A549 Cell Adhesion Assay

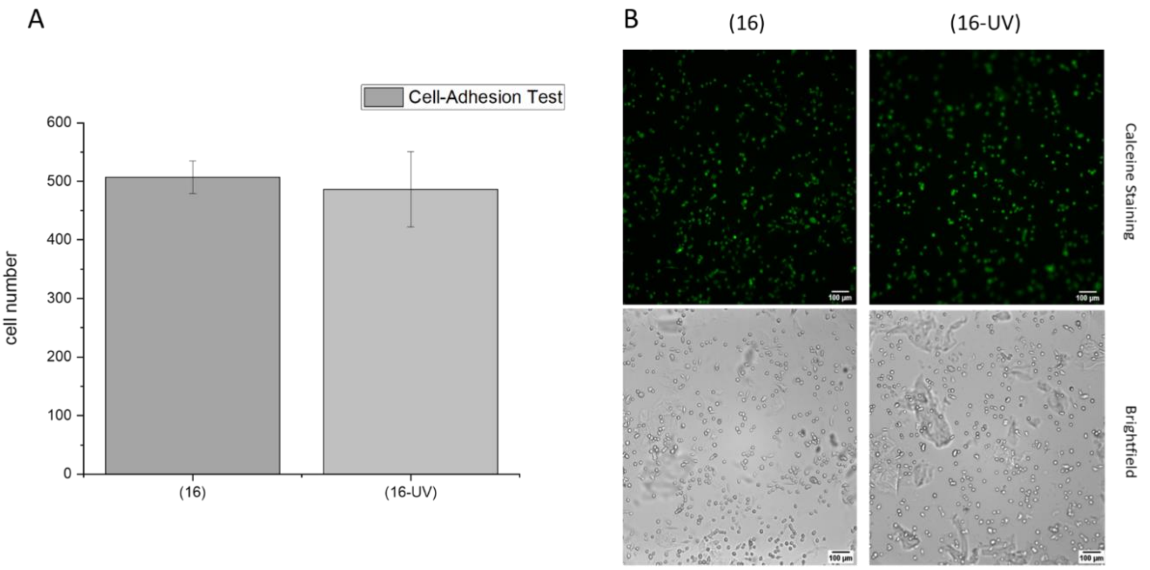

*Figure S11: A549 cell adhesion assay with coated peptide fibers. A) Quantification of cells adhered to substrates coated with **(16)** which was either irradiated **(16-UV)** or left in the dark **(16).** B) Fluorescence images of cells confirming viability through calceine staining and corresponding bright field images. Scalebars represent 100 μm.*

4.9. Cell-Viability Assay

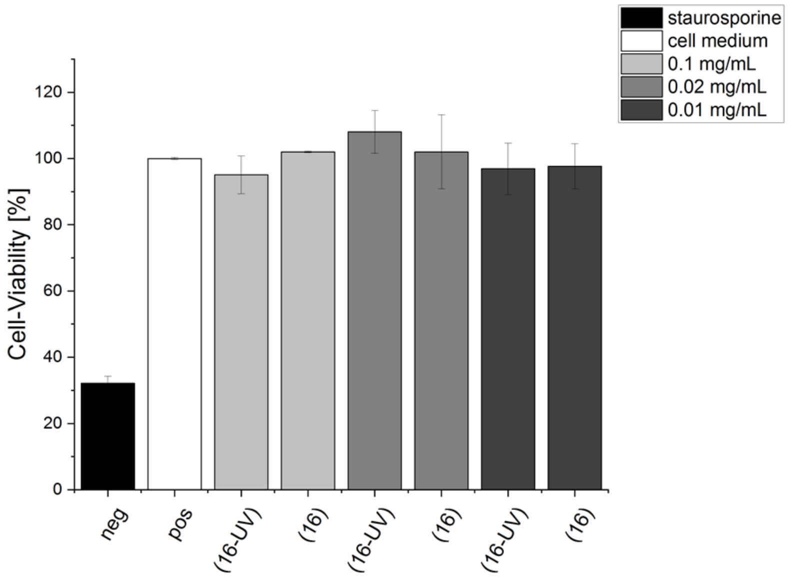

*Figure S12: Cell-Titer-Glo Assay confirms the non-toxic character of the used fibers with **(16-UV)** and without **(16)** irradiation for 10 min. Negative control is a toxic staurosporine solution and positive control are cells without additive.*

5. Additional Characterization RGD-PCL-CKFKFQF **(15)**

5.1. LC-MS

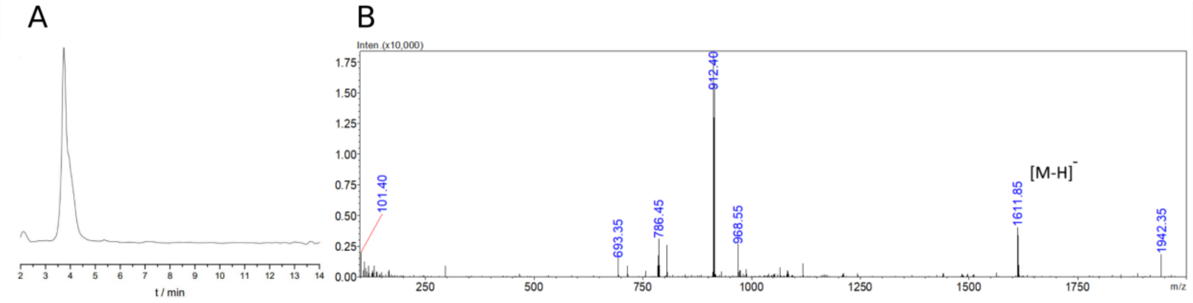

Figure S13: A) LC-MS chromatogram (detector: 214 nm) of RGD-PCL-CKFKFQF ***(15).*** *B) MS (ESI+) m/z calc. for C74H103N18O21S [M-H]-, 1611.73 g mol-1; found 1611.85 m/z*

5.2. A549 Cell Adhesion Assay without Gradient formation

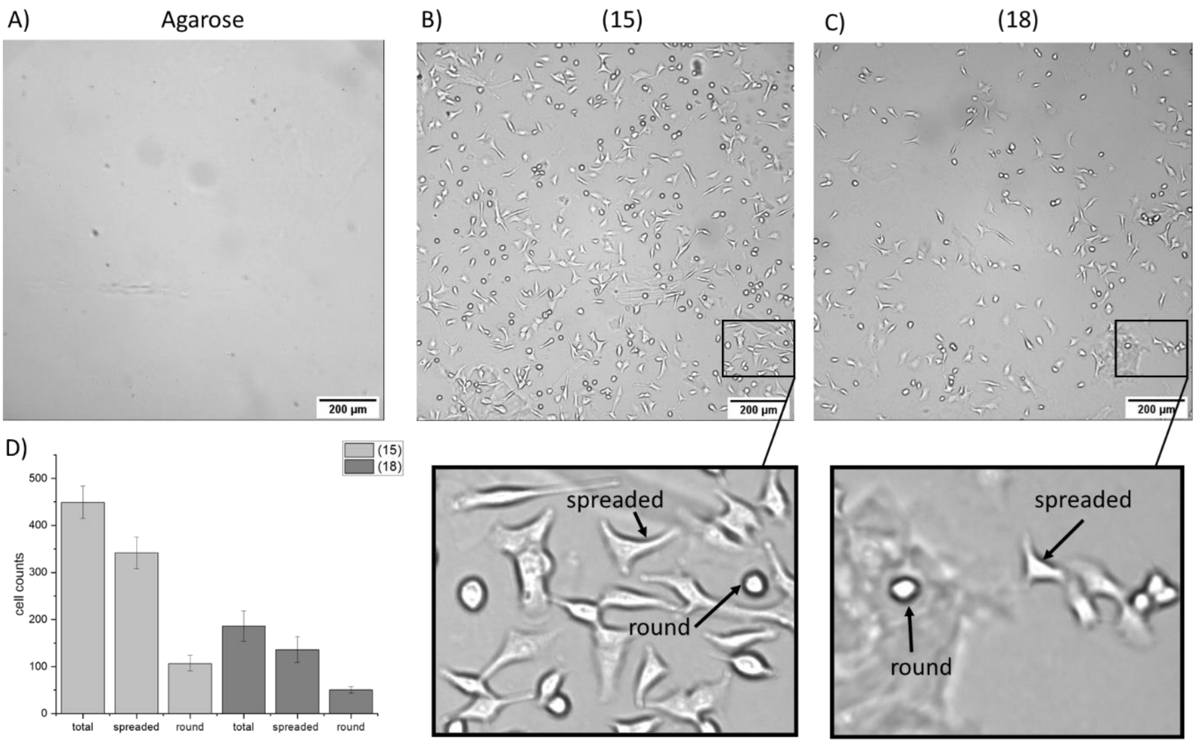

Figure S14: A549 cell adhesion assay. A) Agarose coating prevents adhesion of A549 cells. B) Agarose and peptide fibers ***(15)*** *coated surfaces shows adhesion with cell spreading (see arrow). C) After irradiation* ***(18)*** *less cells are visible on the surface. D) Quantification of cells adhered to substrates coated with* ***(15)*** *which was either irradiated* ***(18)*** *or left in the dark* ***(15).*** *(scalebars: 200 μm)*

5.3. LC-MS Measurements of Non-irradiated Molecule **(15)** and Irradiated Molecule **(18)**

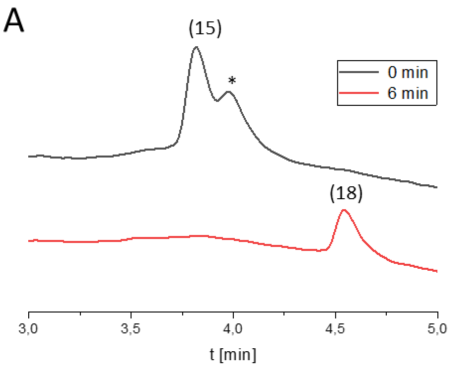

B

N°	t_{Ret} min	$\frac{m}{z}$ (th.)	$\frac{m}{z}$ (exp.)	
		404.2	404	$[M + 4H]^{4+}$
**(15)**	3.8 and 3.9	538.8	538	$[M + 3H]^{3+}$
		807.6	807	$[M + 2H]^{2+}$
		1612.2	1612	$[M + H]^{+}$
**(18)**	4.5	1226.5	1226	$[M + H]^{+}$

*Figure S15: A) LC-MS chromatogram of HPLC fractions (0 sec irradiation for precursor **(15)**) and (6 min irradiation for fragment **(18)**) from the kinetic profiling in HPLC (see main text). Here, the second signal (*) at 4 minutes has the same masses like the first signal **(15)**. The reason for this could be the formation of a disulfide bond between two precursor molecules that was enhanced because of the presence of DMSO in the peptide solution.[3] B) Assignment of signals found in LC-MS measurements of samples before and after UV treatment.*

5.4. Comparison of cleavage kinetics of **(15)**

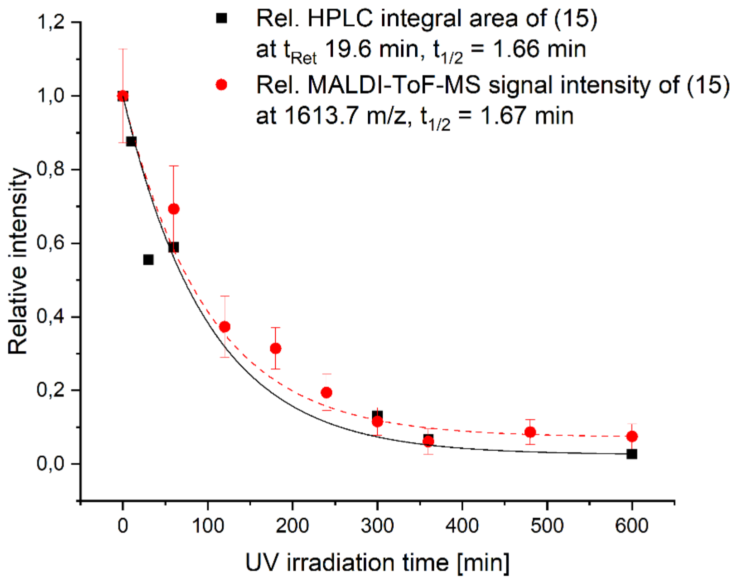

*Figure S16. Relative HPLC integral area of **(15)** at t_{Ret} 19.6 min (black square) and relative MALDI-ToF-MS signal intensities of **(15)** at 1613.7 m/z (red circle) after different UV-treatment times in aqueous solution and in dried state coated on ITO-glass substrate, respectively. Both data were set relative to non-irradiated **(15)** at time 0 and fitted to exponential decay first-order kinetics (solid black line HPLC integral area $t_{1/2}$ = 1.66 min, dashed red line MALDI-ToF-MS $t_{1/2}$ = 1.67 min).*

5.5. Scratch assay of ITO slides coated with (**15**) by atomic force microscopy

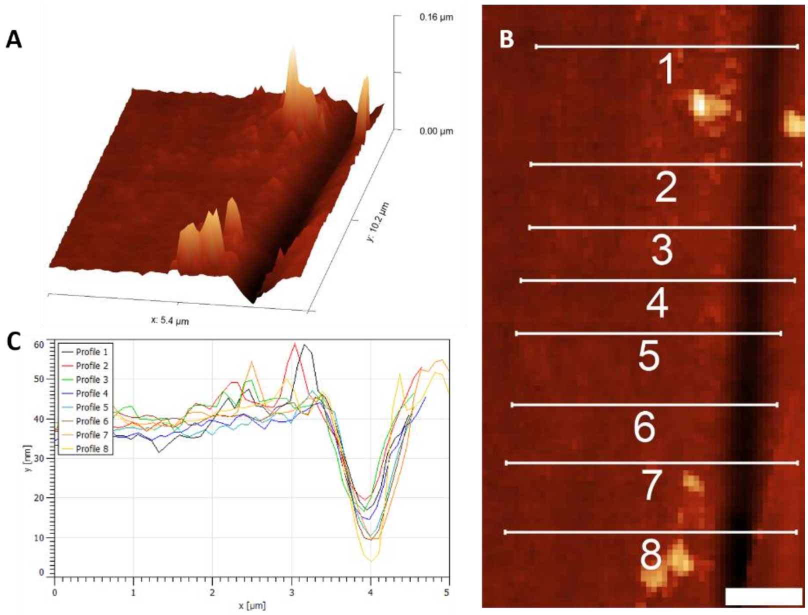

*Figure S17: A) AFM measurement of ITO surface coated with **(15)**. Prior to the measurement, the coating was scratched in order to measure profile thickness. B, C) Along the scratch, 8 profile lines were measured and height differences were calculated between lowest point and 3 µm left from lowest point. The average coating thickness was determined to be 25 ± 5 nm. The scalebar represents 1 µm.*

6. References

(1) Sur, S.; Matson, J. B.; Webber, M. J.; Newcomb, C. J.; Stupp, S. I. Photodynamic Control of Bioactivity in a Nanofiber Matrix. *ACS Nano* **2012**, *6* (12), 10776–10785. https://doi.org/10.1021/nn304101x.

(2) Kobayashi, T.; Seki, T. Odd-Even Effects in Azobenzene-Urea Amphiphile Assemblies. *Langmuir* **2003**, *19* (22), 9297–9304. https://doi.org/10.1021/la035056w.

(3) Papanyan, Z.; Markarian, S. Detection of Oxidation of L-Cysteine by Dimethyl Sulfoxide in Aqueous Solutions by IR Spectroscopy. *J. Appl. Spectrosc.* **2013**, *80* (5), 775–778. https://doi.org/10.1007/s10812-013-9841-1.
